# Cross-kingdom RNA interference in the dysbiosis-mediated network of vocal fold fibrosis: a pathogenic hypothesis and molecular intervention targets

**DOI:** 10.3389/fimmu.2026.1923591

**Published:** 2026-09-15

**Authors:** Quanwei Ren, Yilin Xiang, Di Wu, Yan Bao, Xiaoli Luo, Chao Yu, Yanli Zhao, Xin Zhou

**Affiliations:** Department of Otolaryngology, The First Affiliated Hospital of Chongqing University of Chinese Medicine, Chongqing Traditional Chinese Medicine Hospital, Chongqing, China

**Keywords:** bacterial small RNAs, cross-kingdom RNA interference, laryngeal microbiota, outer membrane vesicles, SMAD7, TGF-β signaling, vocal fold fibrosis

## Abstract

Vocal fold fibrosis is the principal pathological basis of intractable voice disorders. Previous etiological models have attributed vocal fold scarring solely to mechanical trauma and chemical irritation, whereas the local mucosal microbiota of the vocal folds and its cross-kingdom regulation of host gene expression have long received little attention. Integrating interdisciplinary advances, this review proposes a cross-kingdom pathogenic hypothesis: phonotrauma induces laryngeal dysbiosis and disassembly of the vocal fold epithelial tight junctions, and opportunistic pathogens under stress release outer membrane vesicles (OMVs) enriched in bacterial small RNAs (bsRNAs). These nanoscale carriers traverse the damaged mucosal barrier and infiltrate the vocal fold lamina propria; the bsRNAs they carry are recognized through sequence complementarity by host Argonaute proteins and loaded into the RNA-induced silencing complex (RISC), post-transcriptionally silencing Smad7, a key negative regulator of the transforming growth factor-β (TGF-β) signaling pathway, in vocal fold fibroblasts. Post-transcriptional silencing of Smad7 releases the TGF-β/Smad3 pro-fibrotic signaling axis from negative-feedback inhibition, driving the transition of fibroblasts into myofibroblasts and inducing excessive deposition of dense collagen. In parallel, pathogen-associated molecular patterns such as lipopolysaccharide on the OMV surface activate the Toll-like receptor 4 (TLR4)/NF-κB pathway in resident macrophages of the lamina propria; the released pro-inflammatory cytokines and the cross-kingdom RNA interference within fibroblasts reinforce each other, propelling tissue repair toward a terminal fibrotic state. The resulting “mechanical trauma–dysbiosis–cross-kingdom RNA interference–immune activation” network may explain fibrotic progression maintained by persistent laryngeal dysbiosis after the initiating mechanical injury has resolved, and points to new directions for anti-fibrotic strategies based on microbiota modulation and RNA targeting.

## 1 Introduction

Vocal fold fibrosis is the principal pathological basis of intractable voice disorders and commonly arises from iatrogenic trauma such as resection of vocal fold polyps and cysts, from phonotrauma caused by vocal overuse, and from chronic inflammatory stimulation. During fibrotic progression, the normal elastic fibers and hyaluronic acid within the vocal fold lamina propria are replaced by excessively deposited and highly cross-linked type I and type III collagen, leading to loss of the mucosal wave and irreversible impairment of phonation (1). Research over the past several decades has established that the transforming growth factor-β (TGF-β)/Smad signaling axis is the principal pathway driving the transition of vocal fold fibroblasts (VFFs) into myofibroblasts (2), yet targeted agents capable of reversing vocal fold scarring remain unavailable in clinical practice. Although local glucocorticoid injection exerts a degree of anti-fibrotic effect by modulating Smad signaling, it is frequently accompanied by adverse effects such as vocal fold atrophy, indicating that the upstream triggering mechanism of vocal fold fibrosis remains undefined (3). Natural compounds such as asiatic acid have been shown to improve extracellular matrix (ECM) remodeling in vocal fold scarring through activation of Smad7, demonstrating that Smad7, as a negative regulator of the TGF-β pathway, plays an important role in the prevention and treatment of vocal fold fibrosis (4). However, the molecular mechanism by which Smad7-mediated negative regulation—sufficient to restrain fibrotic progression under physiological conditions—becomes persistently inactivated in chronic vocal fold scarring remains to be elucidated. The present article therefore advances a hypothesis-driven theoretical framework, grounded in published evidence, to explain a plausible upstream mechanism for persistent Smad7 inactivation in vocal fold fibrosis, without presenting original experimental work to test the proposed hypothesis. The overall model is illustrated in **Figure 1**.

**Figure 1:**
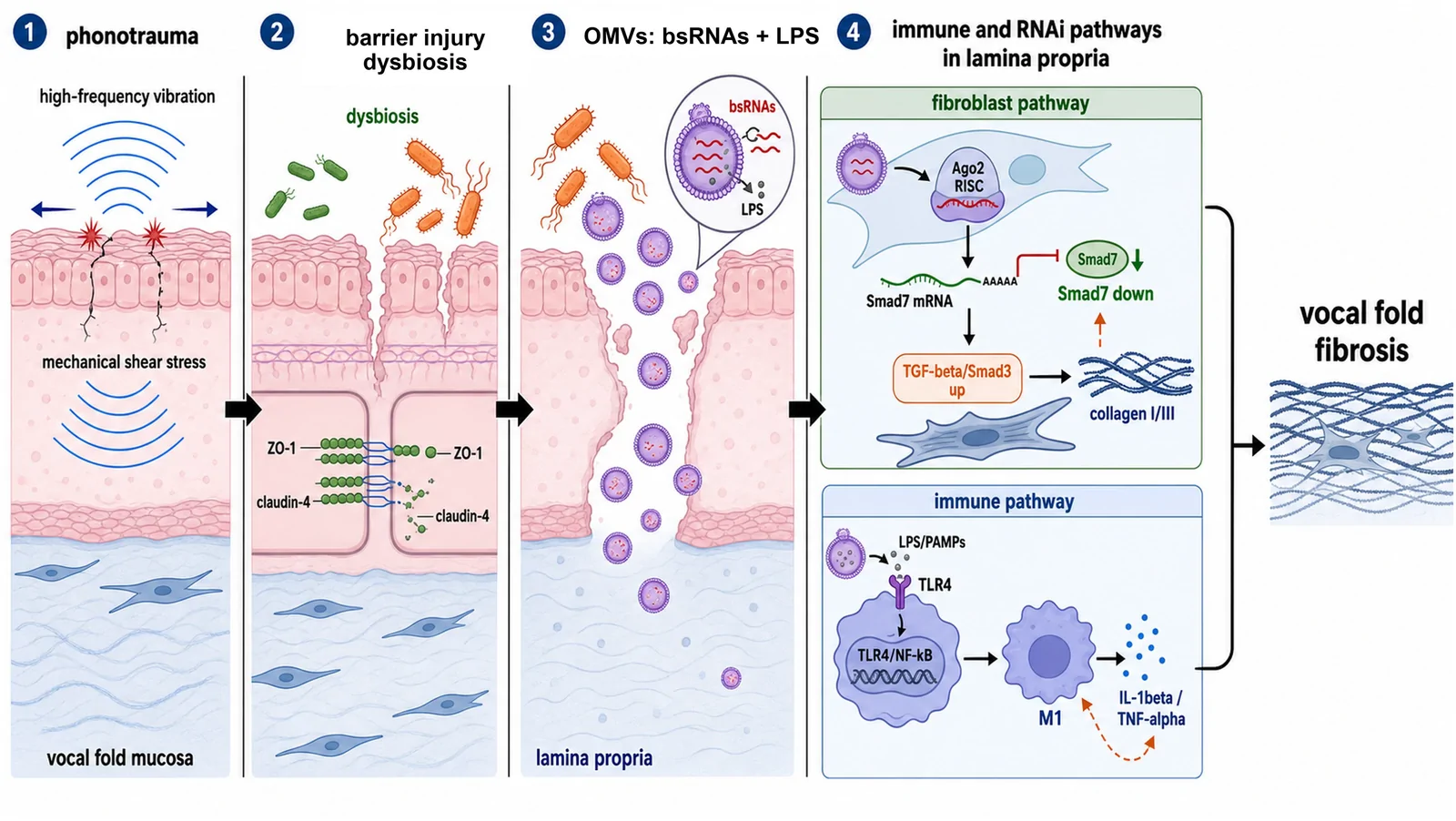
Overall model of vocal fold fibrosis mediated by mechanical trauma, laryngeal dysbiosis, and cross-kingdom RNA interference.

For a long time, etiological research on vocal fold fibrosis has focused mainly on the host’s own physical and chemical stressors, including the shear forces generated by high-frequency vibration and the chemical corrosion of gastric acid reflux. With the development of high-throughput sequencing, however, the important role of the organ microbiome in regulating host immune homeostasis and tissue remodeling has been established. The human larynx and the vocal fold surface were once regarded as a relatively “sterile” transitional region. Current high-throughput sequencing evidence indicates that the laryngeal mucosal surface of healthy individuals harbors a laryngeal microbiome with a defined structure, and that the microbial composition of the laryngotracheal region differs substantially from that of the oral cavity and oropharynx (5). Studies have further proposed the concept of a “gut–larynx axis”, confirming that the resident microbiota of the gut and larynx jointly participate in the dynamic balance of laryngeal immune regulation (6). Under sustained mechanical trauma or inflammatory stimulation, this local microbiota is prone to dysbiosis, manifested as a decline in the abundance of beneficial commensals and the marked proliferation of opportunistic pathogens. Studies of mucosal dysbiosis in patients with idiopathic subglottic stenosis have preliminarily revealed a potential link between laryngeal microbial disturbance and chronic fibrotic inflammation (7). Dysbiosis has been established as an important driver of intestinal fibrosis (e.g., in Crohn’s disease) and pulmonary fibrosis (e.g., idiopathic pulmonary fibrosis), whereas the pathophysiological significance of the laryngeal microbiota in aberrant tissue repair after vocal fold injury remains insufficiently elucidated (8, 9).

In recent years, research has revealed a novel mode of biological communication, namely cross-kingdom RNA interference (ck-RNAi). Pathogens cause disease not only by releasing protein toxins; the nanoscale OMVs they secrete also encapsulate selectively enriched bsRNAs (10). These nanoscale carriers can penetrate the host’s physical barriers and enter human cells, where, mediated by the host’s own RISC, they post-transcriptionally silence host target genes (11). This cross-kingdom gene silencing first gained important experimental support in plant–fungal interaction systems: small RNAs secreted by Botrytis cinerea can be recognized by the Argonaute proteins of the host Arabidopsis thaliana and assembled into functional RISC, silencing plant immunity genes and thereby promoting infection (12). Host plants have also evolved a reciprocal mechanism that delivers their own small RNAs into fungal pathogens via exosomes to silence virulence genes, indicating that vesicle-mediated cross-kingdom delivery of small RNAs is widespread in nature (13). This post-transcriptional regulatory mechanism, which crosses the boundary between prokaryotes and eukaryotes, participates in host immune evasion and chronic inflammatory cascades (14). Further studies indicate that cross-kingdom RNA interference is not confined to plant–fungal interactions. Koeppen et al. observed in a mammalian system that sRNA52320 in Pseudomonas aeruginosa OMVs can be taken up by human airway epithelial cells and functionally downregulate the mRNA levels of the host MAPK-pathway genes MAP2K1 and MAP2K2 (15); the study identified predicted MAPK-pathway targets, but did not perform AGO2-loading assays, AGO2-dependency experiments, or wild-type versus mutant 3′UTR reporter assays. The capacity of plant-derived miRNAs to be delivered across kingdoms into mammalian cells via exosomes and to sequence-specifically silence host target genes has also been confirmed in a series of experiments (16, 17). This evidence from mammalian systems provides the preconditions for the involvement of cross-kingdom RNA interference in the pathogenesis of vocal fold fibrosis.

On the basis of these advances, this review develops a hypothesis-driven theoretical model for the pathogenesis of vocal fold fibrosis: phonotrauma leads to disassembly of the vocal fold epithelial tight junctions and to laryngeal dysbiosis (**Figure 2**), and the bsRNA-enriched OMVs released by opportunistic pathogens subsequently traverse the mucosal barrier and infiltrate the vocal fold lamina propria. Through sequence matching with regions homologous to the host genome, these cross-kingdom small RNA molecules may target and silence Smad7, the negative regulator of the TGF-β pathway, in VFFs, thereby releasing the anti-fibrotic negative-feedback inhibitory mechanism and ultimately leading to excessive matrix deposition. This hypothesis integrates vocal fold–specific evidence—including phonotrauma-induced barrier disruption (19–21), laryngeal dysbiosis (5–7, 24), and the established role of TGF-β/Smad signaling in VFF activation (1–4)—with cross-organ and cross-kingdom mechanistic principles validated in mammalian airway and plant–fungal systems (12, 15). By incorporating the vocal fold microbiota and bacteria-derived small RNAs into the etiological consideration of vocal fold fibrosis, this hypothesis complements the mechanical-injury theory, helps to explain fibrotic progression maintained by persistent laryngeal dysbiosis after the initiating mechanical injury has resolved, and points to new research directions for anti-fibrotic strategies based on microbiota modulation and RNA-targeted therapy. The evidence status of each independent link in the proposed cascade is summarized in **Figure 3**.

**Figure 2:**
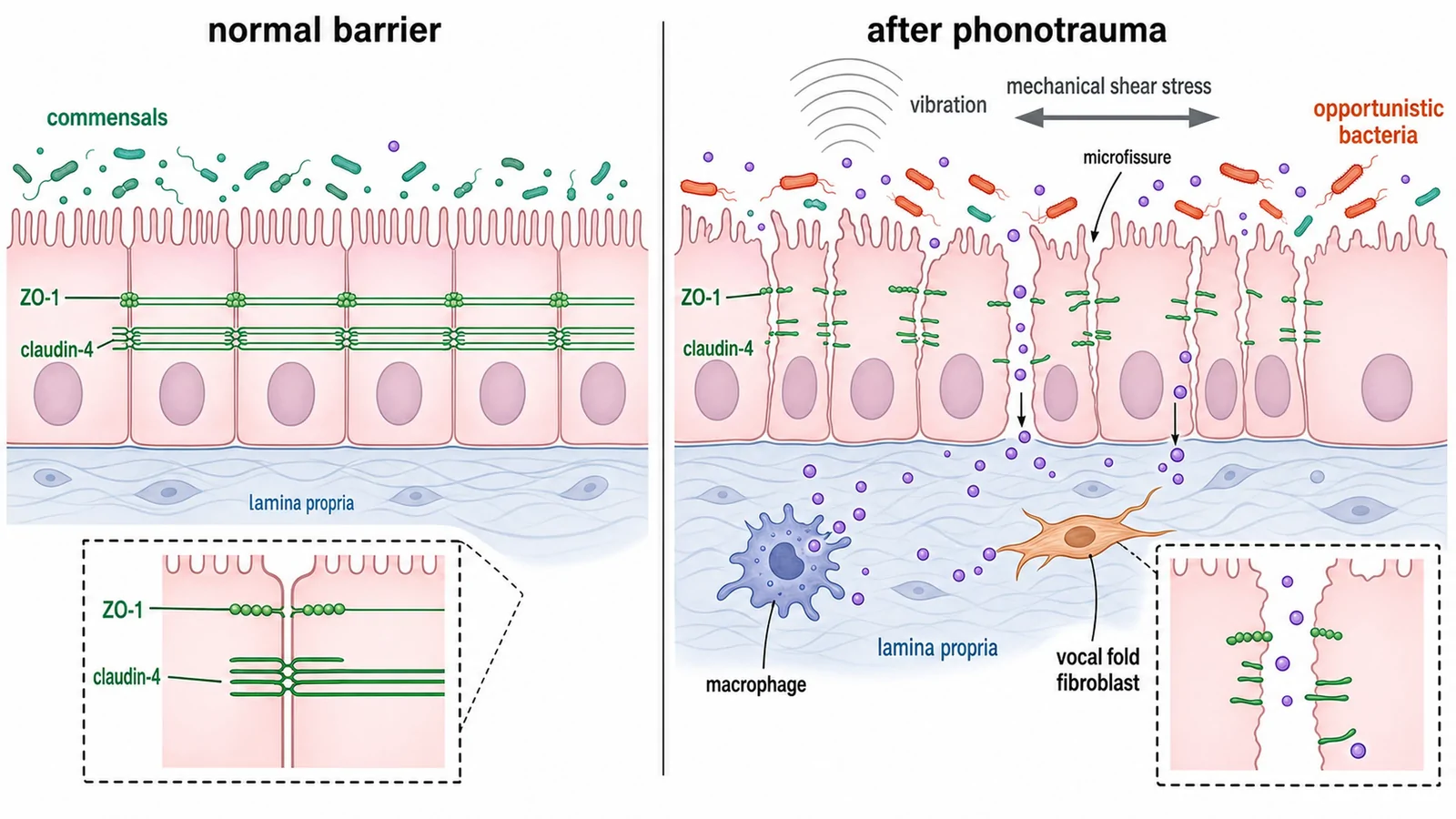
Spatial process of vocal fold epithelial tight-junction disassembly and OMV entry into the lamina propria after phonotrauma.

**Figure 3:**
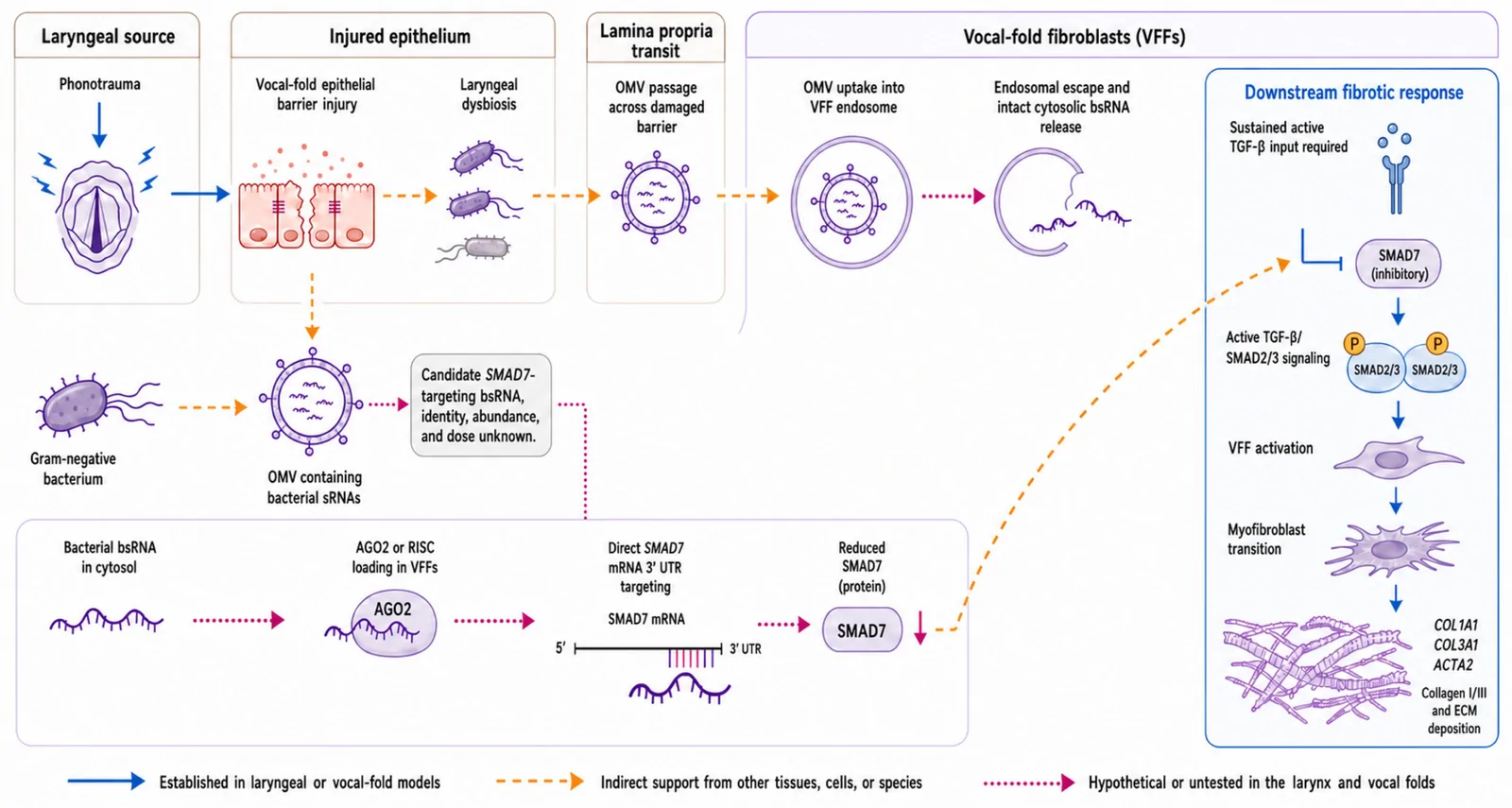
Evidence status of individual links in the proposed laryngeal OMV–bsRNA–SMAD7 cascade.

Solid blue arrows denote relationships established in laryngeal or vocal-fold models, dashed amber arrows denote indirect support from other tissues, cells, or species, and dotted magenta arrows denote links that remain untested in the larynx and vocal folds. Vocal-fold evidence supports phonotrauma-associated epithelial barrier injury and TGF-β/SMAD-mediated VFF activation and ECM deposition. Other biological systems provide indirect support for OMV production, bacterial sRNA cargo, barrier passage, mammalian-cell uptake, and SMAD7-mediated negative regulation. The identity and production of a relevant laryngeal bsRNA, intact cytosolic release in VFFs, AGO2 loading, direct targeting of the SMAD7 mRNA 3′ UTR, and operation of the complete upstream cascade remain unvalidated.

Phonotrauma causes injury to the vocal fold epithelial barrier and laryngeal dysbiosis, and Gram-negative opportunistic pathogens release outer membrane vesicles (OMVs) carrying bacterial small RNAs (bsRNAs) and lipopolysaccharide (LPS). After traversing the damaged epithelium and entering the vocal fold lamina propria, OMVs on the one hand deliver bsRNAs to vocal fold fibroblasts, where, via Ago2/RNA-induced silencing complex (RISC), they target Smad7 mRNA and enhance TGF-β/Smad3 pro-fibrotic signaling; on the other hand, LPS and other pathogen-associated molecular patterns (PAMPs) activate the macrophage TLR4/NF-κB pathway and promote the release of inflammatory cytokines such as IL-1β and TNF-α. The two routes reinforce each other, ultimately leading to collagen I/III deposition and vocal fold fibrosis.

## 2 The biomechanical microenvironment of the vocal fold and the route of pathogen invasion

### 2.1 High-frequency mechanical stress induces disassembly of epithelial tight junctions and creates a route for microbial invasion

During normal phonation, the human vocal folds undergo high-frequency vibration and collision at 100 to 1000 cycles per second, a physical environment that subjects the vocal fold mucosa to substantial shear, tensile, and compressive forces. Under physiological conditions, the stratified squamous epithelium of the vocal folds maintains a physical and immune barrier through tight-junction protein complexes, among which zonula occludens-1 (ZO-1) and the claudin family of proteins are the structural proteins that form the tight-junction scaffold (18). Tight junctions not only act as a physical barrier that restricts the paracellular permeation of luminal contents but also participate in the regulation of epithelial proliferation and survival, constituting an important component of mucosal barrier function. The functional integrity of the vocal fold epithelial barrier in both health and injury is a prerequisite for maintaining the normal vibratory properties of the vocal folds (19). Under vocal overuse or sustained physical microtrauma, excessive mechanical stress induces cytoskeletal remodeling, leading to the depolymerization and degradation of tight-junction structural proteins such as ZO-1 and claudin-4. High-intensity phonation can compromise the integrity of the vocal fold epithelial barrier, and disassembly of the tight-junction complex markedly reduces the transepithelial electrical resistance of the epithelial layer (20). Repair of the vocal fold epithelium after acute phonotrauma follows a defined temporal program, but the cumulative effect of repeated microtrauma can lead to progressive decompensation of the barrier-repair mechanism (21). This disassembly of the tight junctions creates microfissures in the previously dense epithelial layer, producing a “leaky barrier” state that allows luminal microorganisms and their metabolites to invade the deeper lamina propria. Chemical injurious factors such as electronic-cigarette vapor and cigarette smoke can likewise induce vocal fold epithelial injury and trigger mucosal remodeling, aggravating the impairment of barrier function (22, 23). The combined action of these physical and chemical factors provides the structural conditions for the transmucosal infiltration of microbial components.

### 2.2 Mechanical trauma drives the community succession of the laryngeal microbiota from homeostasis to a pathogenic state

The development of high-throughput sequencing has overturned the traditional view of the larynx as “relatively sterile”. The laryngeal mucosal surface of healthy individuals is not sterile but harbors a commensal microbial community with a defined structure and function. These commensals participate in the maintenance of local mucosal immune homeostasis—that is, in maintaining physiological low-grade immune tolerance toward commensals while preserving the capacity to defend against invading pathogens. Phonotrauma disrupts the physical barrier and induces local microvascular ischemia and hypoxia as well as abrupt changes in the pH of the tissue microenvironment, leading to laryngeal dysbiosis. A systematic review has confirmed that the laryngotracheal region possesses a microbial composition markedly different from that of the oral cavity and oropharynx, and that dysbiosis in these regions is closely associated with a variety of upper respiratory tract diseases (5). Within the “gut–larynx axis” framework, the resident laryngeal microbiota interacts with the host immune system through metabolites and immune signaling molecules to maintain immune homeostasis at the mucosal surface (6). Under the sustained drive of mechanical trauma and chronic inflammation, the laryngeal microbiota undergoes community succession from homeostasis to a pathogenic state: local microbial diversity declines, the abundance of protective probiotics decreases, and the relative abundance of Gram-negative opportunistic pathogens such as *Fusobacterium nucleatum* and *Pseudomonas aeruginosa* increases. “Laryngeal opportunistic pathogens” refers to Gram-negative genera that are present at low relative abundance in the healthy laryngeal mucosal microbiota but undergo overgrowth and exert pathogenic effects upon barrier injury or dysbiosis. These Gram-negative genera are the expected sources of pathogenic OMVs in this hypothesis. Among them, *Fusobacterium nucleatum* accounts for approximately 15% of the healthy laryngeal microbiota on average and is further enriched in vocal fold lesions (74), and evidence that its OMVs carry RNA cargo and modulate host-cell function has been obtained in multiple tissue models (75). Idiopathic subglottic stenosis is a laryngeal disease characterized by persistent mucosal inflammation and fibrosis; its epithelial dysbiosis is closely associated with disease progression and represents preliminary clinical evidence that laryngeal dysbiosis drives fibrotic lesions (7). Recent studies have confirmed that common laryngeal microbiota such as the genus *Streptococcus* can affect the integrity and barrier function of the vocal fold mucosa, and that colonization by *Streptococcus pseudopneumoniae* is associated with loosening of the intercellular junctions of vocal fold epithelial cells (24). These findings indicate that laryngeal dysbiosis is directly involved in the regulation of vocal fold barrier function. Studies of microbial disturbance in laryngopharyngeal reflux disease further demonstrate a mutually reinforcing relationship between alterations in the laryngeal microenvironment and disruption of the microbial community structure (25).

### 2.3 Bacterial outer membrane vesicles exploit their nanoscale size to cross the disassembled barrier and infiltrate the lamina propria

Under dysbiotic-stress conditions characterized by reduced microbial diversity, depletion of protective commensals, and enrichment of opportunistic pathogens, Gram-negative opportunistic pathogens secrete OMVs ranging from 20 to 250 nm in size. OMVs are bilayered spherical nanostructures actively released by Gram-negative bacteria during growth or stress; the molecules they encapsulate include virulence factors, lipopolysaccharide (LPS), nucleic acids, and various metabolic enzymes, and their functions span interbacterial communication, DNA transfer, and host immune regulation (26). It should be noted that Gram-positive bacteria also release membrane vesicles, but these lack an outer membrane and lipopolysaccharide, differing significantly in structure and composition from Gram-negative OMVs; the present hypothesis focuses on OMVs of Gram-negative origin. Gram-negative bacteria (including the Bacteroidetes, Fusobacteria, and Proteobacteria phyla) constitute approximately 30% of the laryngeal microbiota, a relatively high proportion among respiratory mucosal sites (74), providing a microbial basis for local OMV production. “Pathogenic OMV” is defined in this review as an OMV produced by Gram-negative opportunistic pathogens under dysbiotic-stress conditions that carries molecular cargo—including bsRNA, LPS, and virulence factors—capable of interfering with host-cell function. Because OMVs are continuously produced by diverse Gram-negative bacteria during infection, they serve as intermediaries in host–pathogen interactions (27). The role of gut microbiota-derived OMVs in regulating intestinal mucosal homeostasis has been established, and the OMVs released by *Akkermansia muciniphila* interact with host cells through multiple routes and modulate local immune responses (28). The pathogenic capacity of OMVs derives from their nanoscale size and from their lipid composition, which resembles that of mammalian cell membranes. These features enable OMVs to bypass the mucociliary clearance system at the mucosal surface and to enter deep tissue along the microfissures of the disassembled tight junctions. Molecular dynamics simulation studies have revealed the molecular determinants of OMV internalization by host cell membranes and confirmed that OMVs deliver their molecular cargo directly from the bacterial surface into host target cells through multiple routes (29). The OMVs of *Pseudomonas aeruginosa* can simultaneously deliver multiple virulence factors over long distances into the host cytoplasm, bypassing conventional secretion-system routes (30). Experimental evidence has shown that the extracellular RNA within the OMVs of the periodontal pathogen *Aggregatibacter actinomycetemcomitans* can promote the production of tumor necrosis factor-α (TNF-α) by human macrophages and, in a mouse model, can cross the blood–brain barrier and enter brain tissue (31), indicating that OMV-borne RNA molecules can cross intact biological barriers and exert functional effects in distant organs. After entering the vocal fold lamina propria, OMVs carrying pathogen-associated molecular patterns (PAMPs) and nucleic acids come into contact with resident macrophages and fibroblasts within the matrix, constituting the intermediary of molecular transfer between bacteria and host. It should be noted that the vocal fold is a low-microbial-biomass tissue; bacterial DNA concentrations in the mouse larynx are approximately 10–40 ng/μL, far lower than those in the gut (>600 ng/μL), and quantitative data on *in vivo* OMV production in the larynx are currently lacking. OMV production by Gram-negative bacteria increases significantly under host-related stress conditions (76), and gut-derived OMVs have been shown to cross the epithelial barrier and enter the circulation, reaching concentrations of 10^7^–10^10^ particles/mL in blood (76). OMVs can selectively enrich bsRNAs (15). Quantitative EV studies indicate that cytosolic release of RNA cargo is limited and varies with vesicle source, recipient cell, and assay method (77). The actual cytosolic delivery fraction of laryngeal OMV-bsRNA has not been directly quantified and may be jointly determined by local bacterial load, OMV production, barrier-injury severity, and vesicle-clearance rate. Once in the recipient-cell cytosol, exogenous bsRNAs must share a limited AGO-loading system with endogenous miRNAs. Absolute quantification in mouse embryonic stem cells indicates that cellular miRNA abundance spans several orders of magnitude and that silencing depends on RNA abundance, AGO loading, and miRNA-to-target-pool stoichiometry (81). The effective cytosolic copy number of a candidate bsRNA is therefore a key determinant of quantitative plausibility, and its AGO2-loading fraction further determines how many copies form active silencing complexes.

Under physiological conditions, vocal fold epithelial cells are tightly arranged, tight-junction proteins such as ZO-1 and claudin-4 maintain a continuous barrier structure, and the commensal microbiota remains mainly on the luminal surface. After phonotrauma, mechanical shear forces cause loosening of the intercellular junctions, rupture of tight-junction proteins, and the formation of microfissures. Following laryngeal dysbiosis, opportunistic pathogens proliferate and release OMVs of 20 to 250 nm. OMVs enter the lamina propria along the damaged barrier and come into contact with resident macrophages and vocal fold fibroblasts, providing the spatial conditions for subsequent immune activation and cross-kingdom RNA interference.

## 3 A Pathogenic hypothesis: cross-kingdom small RNAs target and remodel the pro-fibrotic network

### 3.1 OMV-mediated cross-kingdom delivery of bacterial small RNAs constitutes the material basis of cross-kingdom gene regulation

After entering the vocal fold lamina propria, OMVs act as exogenous antigens that activate immune cells and are presumed to be taken up—via endocytosis, macropinocytosis, or direct membrane fusion—by VFFs, the principal effector cells of the vocal fold lamina propria (29); this uptake mechanism is inferred from evidence in other cell types, and the specific mechanism by which VFFs internalize OMVs remains to be verified. OMVs secreted by *Neisseria gonorrhoeae* can be efficiently endocytosed by epithelial cells, releasing functional molecules intracellularly and inducing metabolic reprogramming of the host cells (32). Multi-omics sequencing studies have confirmed that OMVs are internally enriched, under *in vitro* culture conditions, with selectively packaged bsRNAs whose actual abundance in the *in vivo* vocal fold microenvironment remains to be verified. OMV RNA includes independently transcribed bacterial regulatory sRNAs, short RNAs processed from longer transcripts by RNase E or RNase III, and tRNA/rRNA-derived fragments with reproducible 5′ ends, 3′ ends, and dominant lengths across independent biological replicates. Independently transcribed bacterial sRNAs commonly terminate at Rho-independent terminators and do not follow the Drosha/Dicer biogenesis pathway of eukaryotic miRNAs; RNA origin or a length of 15–50 nt alone does not establish cross-kingdom regulatory function (10, 33). Candidate cross-kingdom regulatory bsRNAs are therefore defined here as bacterial RNAs that are stably and selectively enriched in OMVs, reach the host cytosol, are compatible with host AGO loading, and have sequence-specific host-targeting potential. Studies have confirmed that bacteria-derived extracellular vesicles contain small RNAs and transfer RNAs capable of regulating the inflammatory response in pulmonary infection (33).

A study by Koeppen et al., published in PLoS Pathogens in 2016, showed that sRNA52320, a small RNA differentially packaged in the OMVs of Pseudomonas aeruginosa, can be taken up by human airway epithelial cells and functionally downregulate the mRNA levels of mitogen-activated protein kinase kinase 1 (MAP2K1) and MAP2K2 within host cells (15). The study identified predicted MAPK-pathway targets and observed functional effects, but did not prove direct binding of sRNA52320 to specific MAPK transcripts, nor did it perform AGO2-loading assays, AGO2-dependency experiments, or wild-type versus mutant 3′UTR reporter assays. This was the first experimental study to show in a mammalian system that OMV-borne bacterial sRNA can functionally regulate host target-gene expression, advancing OMV-sRNA-mediated host gene regulation from theoretical inference in the field of plant–fungal interactions to the initial empirical level in mammalian systems. Salmonella Sal-1 is derived from the 5′ leader of an rRNA transcript and is processed through an approximately 74-nt stem-loop precursor into a 24-nt mature fragment; its maturation depends on host AGO2 rather than Dicer, providing direct evidence for a non-canonical host AGO2-associated processing route for bacterial RNA (34). The AGO2 MID domain anchors the 5′ monophosphate of a guide RNA and preferentially recognizes 5′U/A over 5′C/G (82); approximately 21–23-nt duplexes bearing a 5′ monophosphate and 2-nt 3′ overhangs are canonical structural features for loading miRNA/siRNA duplexes into human AGO proteins (84). These features define biochemical compatibility criteria, whereas actual loading must be tested by end-chemistry analysis and AGO2-binding assays. A study published in Nature in 2023 by Cao et al. further confirmed that small RNAs of Pseudomonas aeruginosa regulate the switch between chronic and acute infection lifestyles in human-derived samples, indicating that bacterial sRNAs are functionally expressed within the human host (35). In a Legionella pneumophila–mammalian cell system, the regions at nucleotides 48–65 of RsmY and 59–73 of tRNA-Phe that resemble host miRNAs were associated with targeting effects on the 3′UTRs of host ddx58 (RIG-I), irak1, and cRel. Luciferase assays supported the corresponding targeting effects, and AGO2 inhibition implicated RISC in this regulation (72). These similarity regions are approximately 18 nt and 15 nt, respectively; the natural RNA species, exact termini, and AGO2-loading forms that act in host cells remain to be determined. Sal-1 provides the strongest precedent for AGO2-dependent processing, whereas sRNA52320 is a 24-nt tRNA-Met-derived fragment with selective OMV enrichment and host-delivery effects but a 5′C and no direct AGO2-loading evidence (15, 34). These molecules satisfy different subsets of the proposed criteria and provide priority reference classes for candidate cross-kingdom regulatory bsRNAs. Wu et al. demonstrated that Gram-negative OMV-sRNAs suppress host defense genes in a plant system, providing plant-level experimental evidence for OMV-mediated cross-kingdom delivery of bacterial sRNAs (73).

Several independent studies have confirmed that plant-derived miRNAs can enter mammalian cells through exosome-like nanoparticle-mediated cross-kingdom delivery and sequence-specifically silence host target genes. Plant-derived miR166a-3p can be delivered across kingdoms into mammalian mammary cells via exosomal packaging, inhibiting cell proliferation and promoting apoptosis by targeting the APLNR gene (16). Reviews have further noted that cross-kingdom gene regulation is a biological mechanism widespread in nature and that plant-derived small RNAs can exert regulatory functions of therapeutic value within mammals (17, 36). Studies on the bioactivity of miRNAs in plant-derived exosome-like nanoparticles also provide technical support for vesicle-mediated cross-kingdom RNA delivery (37). Although the concept of exogenous miRNAs regulating mammalian genes across kingdoms remains controversial (38, 39), an increasing body of experimental evidence indicates that, under specific conditions—particularly vesicle-mediated protective delivery—cross-kingdom sRNA-mediated host gene regulation is biologically plausible in mammalian systems.

If OMVs complete endosomal escape and release their RNA cargo into the cytoplasm of host fibroblasts—a step for which direct experimental evidence in mammalian cells is currently lacking, as fluorescent labeling can only confirm OMV uptake and does not prove intact cytosolic release available for AGO2 loading—candidate bsRNAs with reproducible 5′ ends, 3′ ends, and dominant lengths across independent biological replicates and with host-AGO-compatible features may enter the host gene-regulatory network as post-transcriptional regulators. Cross-kingdom RNA interference is regarded as a mode of communication between organisms, in which sRNA molecules can be transferred between different kingdoms to silence specific genes (14). The sRNAs of *Botrytis cinerea* are recognized by the Argonaute proteins of the host *Arabidopsis thaliana* and assembled into functional RISC, capable of silencing plant immunity genes (12). The reciprocal mechanism by which host plants deliver sRNAs into fungal pathogens via exosomes to silence virulence genes further indicates that vesicle-mediated cross-kingdom delivery of sRNAs is an evolutionarily conserved mode of signal transmission in nature (13).

### 3.2 Smad7, the natural negative regulator of the TGF-β/Smad signaling axis, is a key node in vocal fold anti-fibrosis

During physiological repair after vocal fold mucosal injury, TGF-β1 is the driver that initiates fibroblast activation and ECM synthesis (2). As the foremost regulator of the fibrotic process, TGF-β1 signaling drives scar formation in a variety of chronic tissue injuries (40). After TGF-β1 binds its specific receptor, it triggers a cascade of phosphorylation of Smad2/3 proteins; the activated Smad2/3 complex translocates into the nucleus and drives the transcription of fibrotic genes such as type I and type III collagen (41). RNA-sequencing studies have revealed the transcriptomic differences between VFFs and myofibroblasts, the latter characterized mainly by the abundant secretion of ECM components and the assembly of stress fibers (42). Changes in matrix adhesiveness and in the mechanical properties of the microenvironment can regulate the differentiation of VFFs into myofibroblasts, with activation of the MRTF-A/SRF signaling axis serving as the downstream effector that drives this pathological phenotypic transition (43, 44). To prevent repair from progressing to excessive connective-tissue proliferation, the host system has evolved a negative-feedback inhibitory mechanism, namely the inhibitory Smads, among which Smad7 performs the principal negative-regulatory function (45). By competitively binding the TGF-β type I receptor and recruiting the ubiquitin ligases Smurf1/2 to mediate ubiquitination and degradation of the receptor, Smad7 blocks the excessive amplification of the pro-fibrotic cascade at the upstream end of signaling (45). In vocal fold fibrosis, the evidence that asiatic acid improves ECM remodeling in vocal fold scarring by activating Smad7 further corroborates the role of Smad7 in maintaining the homeostasis of vocal fold tissue repair (4). Under physiological conditions, the inducible expression of Smad7 is sufficient to restrain excessive activation of the TGF-β/Smad3 signaling axis, keeping tissue repair at a reversible, adaptive stage. The status of Smad7 as a negative regulator of the TGF-β signaling pathway has been validated across organs. Reviews have confirmed that Smad7 continuously exerts negative-regulatory functions in health and disease and that its dysfunction is associated with a variety of fibrotic and inflammatory diseases (46). In cardiac fibrosis models, induced expression of Smad7 in fibroblasts protects the pressure-overloaded heart from excessive fibrosis and adverse remodeling (47). By simultaneously restraining the TGF-β and ErbB2 signaling pathways, Smad7 inhibits excessive myofibroblast activation and protects cardiac function after myocardial infarction, whereas Smad7 deficiency leads to uncontrolled fibrosis and worsening heart failure (48). This cross-organ evidence indicates that the functional integrity of Smad7 is a key element in preventing fibroblasts from entering a persistently activated state. Smad7 is the principal negative regulator that restrains vocal fold fibrosis, and any mechanism that causes the loss of Smad7 expression or function will release its negative-feedback inhibition and promote the fibrotic process.

### 3.3 Cross-kingdom RNA interference silences Smad7 and drives the irreversible progression of vocal fold fibrosis

Building on the cross-kingdom interaction theory described above, this review proposes—and it must be emphasized that this remains a hypothesis requiring experimental validation—a molecular pathogenic hypothesis: the specific bsRNAs released by the pathogenic OMVs that arise from dysbiosis may carry sequences partially complementary to the 3’ untranslated region (3’UTR) of human *Smad7* mRNA; the hypothesis posits that these exogenous bacterial RNAs can be recognized and loaded by the Argonaute proteins of human cells, forming a RISC with cross-kingdom silencing capacity (**Figure 4**). Mammalian cells possess a functionally intact RNAi machinery, in which Argonaute proteins act as effectors that achieve sequence-specific target-gene silencing under the guidance of small RNAs (49). AGO2–small RNA complexes can post-transcriptionally silence target genes through translational repression and mRNA destabilization (50). Endogenous miR-21 provides a direct mammalian precedent by targeting the Smad7 3′UTR and repressing its translation (80). Within the framework of this hypothesis, the silencing complex is postulated to post-transcriptionally suppress Smad7 expression within VFFs, leading to a reduction in the inhibitory Smad7 protein. If the natural anti-fibrotic negative-feedback mechanism is thereby inactivated, the TGF-β/Smad3 signaling axis may remain persistently activated owing to the loss of negative regulation, maintaining fibroblasts in the persistently activated pathological myofibroblast phenotype and ultimately potentially inducing excessive deposition of dense collagen and vocal fold scar remodeling. Persistent activation of the TGF-β/Smad3 signaling axis depends on a stable supply of active TGF-β ligand, the sources of which include tissue TGF-β release induced by macrophage pro-inflammatory cytokines and an autocrine TGF-β1 positive-feedback loop in myofibroblasts (78); the role of Smad7 loss in this process is to amplify ongoing signaling rather than to autonomously generate upstream ligand, and its de-repression of the NF-κB pathway further intensifies inflammation-driven TGF-β supply (79). A pathophysiological review of vocal fold fibrosis notes that the irreversible transition of VFFs into myofibroblasts and the excessive deposition of ECM are the cytological endpoints of vocal fold scar formation (51). This hypothesis can also explain the phenomenon whereby vocal fold scarring continues to progress after the initial mechanical injury and acute inflammation have resolved: If laryngeal dysbiosis persists and continuously releases pathogenic OMVs, the post-transcriptional silencing of Smad7 may be sustained, arresting the tissue-repair process in a fibrotic state. Phonotrauma initiates barrier injury and microbial disruption; after the original mechanical injury has healed, persistent laryngeal dysbiosis and OMV release maintain cross-kingdom RNA interference, inflammatory signaling, and fibrotic progression. This persistent-stimulus maintenance mechanism differs from inflammatory memory retained through sustained chromatin accessibility after withdrawal of the initiating stimulus (83). It should be noted that this proposed cascade remains entirely hypothetical at the present stage and requires stepwise experimental validation, as detailed in Section 5.2.

**Figure 4:**
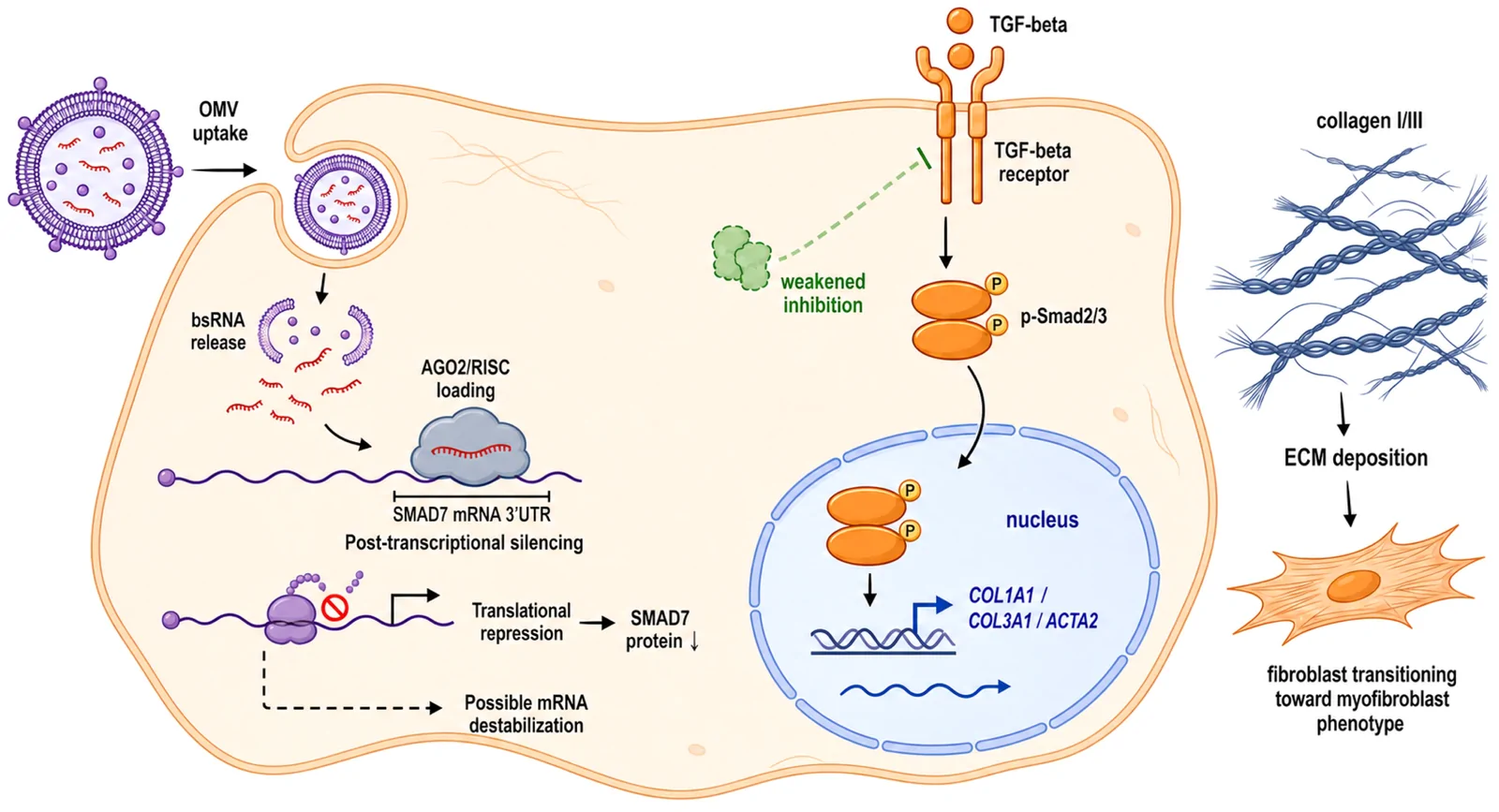
Mechanism of cross-kingdom RNA interference in which bacterial small RNAs, after loading onto Ago2/RISC, target Smad7 mRNA.

After pathogenic OMVs are taken up by vocal fold fibroblasts, they release bsRNAs into the cytoplasm. Once loaded onto Ago2/RISC, the bsRNAs bind through sequence complementarity to the 3’UTR region of Smad7 mRNA, post-transcriptionally suppressing Smad7 expression and reducing the Smad7 protein level. With the attenuated negative regulation of the TGF-β receptor by Smad7, Smad2/3 phosphorylation and nuclear translocation increase, and the expression of pro-fibrotic genes such as COL1A1, COL3A1, and ACTA2 is enhanced, ultimately promoting the phenotypic transition of vocal fold fibroblasts into myofibroblasts and causing extracellular matrix (ECM) deposition.

## 4 Synergistic amplification by the local immune microenvironment

### 4.1 Pathogen-associated molecular patterns on the OMV surface drive M1 polarization of resident macrophages and the release of pro-inflammatory cytokines

In addition to the aberrant post-transcriptional silencing within fibroblasts, the OMVs that infiltrate the lamina propria also constitute an exogenous immune-activating signal. A certain number of macrophages responsible for immune surveillance reside within the human vocal fold matrix. Microbial structural molecules such as the LPS embedded on the OMV surface can be recognized by Toll-like receptor 4 (TLR4) on the surface of these macrophages. The mechanism by which OMVs mediate the entry of LPS into the host cytoplasm and activate the caspase-11 inflammasome was confirmed by Vanaja et al., revealing the immunological function of OMVs as carriers that deliver PAMPs intracellularly (52). Studies showing that the OMVs of *Porphyromonas gingivalis* redirect host innate immunity and promote inflammation through their lipid A component further confirm the role of OMV surface molecular patterns in regulating the direction of the host immune response (27). During vocal fold fibrosis, activation of the TLR4 receptor triggers an intracellular signal-transduction cascade in macrophages, leading to nuclear translocation of the NF-κB complex. Urban particulate matter can promote vocal fold fibrosis through the MAPK/NF-κB signaling pathway, corroborating the functional activity of this inflammatory pathway in vocal fold tissue (53). Nuclear translocation of NF-κB drives the polarization of resting macrophages toward the M1 pro-inflammatory phenotype and the rapid release of pro-inflammatory cytokines such as interleukin-1β (IL-1β) and TNF-α (54).

### 4.2 Immune inflammation and cross-kingdom RNA interference cooperatively drive the irreversible progression of fibrosis

The local inflammatory response and the cross-kingdom RNA interference within fibroblasts reinforce each other. On the one hand, the inflammatory factors released by macrophages recruit peripheral monocytes to the injury site, expanding the local pool of inflammatory cells; on the other hand, they continuously stimulate the injured mucosal tissue to release activated TGF-β molecules, replenishing the ligand source for the sustained activation of fibroblasts (40). Within the framework of this hypothesis, VFFs are thus simultaneously acted upon by two routes: the pro-fibrotic stimulation of inflammatory mediators and the hypothesized bsRNA-mediated inactivation of Smad7 function. Under this dual action, the originally reversible tissue edema and mild hyperplasia progress to dense fibrosis, impairing the viscoelasticity of the vocal fold tissue. Studies of radiation-induced vocal fold fibrosis further confirm that sustained inflammatory drive and ECM deposition constitute a terminal pathological pathway shared by multiple etiologies (55). These interactions explain a clinical feature of vocal fold fibrosis: after the initiating mechanical or chemical insult has resolved, lesion progression may be jointly maintained by persistent laryngeal dysbiosis, continued OMV input, and the tissue-intrinsic “inflammatory cytokine secretion–TGF-β activation” positive-feedback loop. While this positive-feedback model is mechanistically plausible, direct experimental evidence for its operation in vocal fold tissue remains to be established.

## 5 Discussion and perspectives

### 5.1 Potential directions for noninvasive diagnostic biomarkers of vocal fold fibrosis

Incorporating outer membrane vesicles and the laryngeal microbiota into the analysis of pathogenesis suggests new directions for the noninvasive molecular diagnosis of vocal fold fibrosis. Compared with conventional stroboscopic diagnostic techniques that rely on morphological observation, future clinical practice may use minimally invasive sampling of laryngeal surface mucus or saliva and apply high-throughput sequencing or liquid biopsy to monitor the titer of locally characteristic pathogen-derived OMVs, or to directly quantify the abundance of characteristic bsRNAs with potential pathogenic risk (8). The microbial composition of the laryngotracheal region is associated with a variety of upper respiratory tract diseases, and these microbe-derived components may serve as key early-warning markers for assessing the evolution of simple vocal fold injury toward scarring (5). Spatial transcriptomics has revealed the spatial distribution of fibroblast niches and intercellular communication networks in studies of the pathological microenvironment of idiopathic pulmonary fibrosis (56) and is equally applicable to the study of vocal fold fibrosis. A multi-omics strategy that integrates single-cell and spatial transcriptomics can resolve the interaction map of “microbial metabolites–host cell populations” at both single-cell resolution and spatial location, helping to elucidate the dynamic intercellular communication during the temporal evolution of vocal fold fibrosis (57, 58).

### 5.2 Validation pathway and technical feasibility of the cross-kingdom RNA interference hypothesis

Koeppen et al. have demonstrated that OMV-sRNA can downregulate host MAPK genes in human airway epithelial cells (15). By applying the same OMV–host cell co-culture system, replacing the target gene with Smad7 and the host cells with VFFs, the silencing effect of OMV-bsRNA on Smad7 mRNA can be examined. With regard to *in vitro* platforms, vocal fold biomechanics research has established a dynamic culture system in which VFFs are cultured in a microenvironment simulating high-frequency vibration, reproducing the mechanotransduction signals that the vocal folds experience under physiological phonation (59). Combining such a dynamic mechanical culture system with an OMV co-culture system would allow the construction of an *in vitro* model that recapitulates the complete cascade of “barrier injury–microbial infiltration–fibroblast response” after vocal fold microtrauma. Although sampling and microbial analysis of the laryngeal microbiota face technical difficulties such as small sample size (25), the combined strategy of laryngeal brush sampling with 16S rRNA amplicon sequencing and metatranscriptomics has been successful in laryngotracheal microbiota research (5), and these technical approaches can be directly applied to the analysis of characteristic microbial profiles in cohorts of patients with vocal fold fibrosis. For the functional validation of this hypothesis, current technologies provide a clear stepwise validation pathway (**Figure 5**). First, the OMV-sRNA repertoire of laryngeal opportunistic pathogens will be screened against the Smad7 3′UTR. Candidate prioritization will assess reproducibility of 5′ ends, 3′ ends, and dominant lengths across biological replicates; 5′-phosphorylation status and terminal-nucleotide preference; duplex and 3′-end structure; selective OMV enrichment; and cytosolic delivery, thereby identifying candidates compatible with host AGO2 loading. Second, OMVs can be isolated from these pathogens cultured under stress conditions mimicking the dysbiotic microenvironment, characterized by nanoparticle tracking analysis and transmission electron microscopy, and their bsRNA cargo confirmed by small RNA sequencing. Third, co-culture of purified OMVs with primary human VFFs, combined with fluorescent lipid labeling to quantify uptake and RT-qPCR/Western blot to measure Smad7 mRNA and protein levels, can directly test whether OMV exposure reduces Smad7 expression. Fourth, AGO2-RNA immunoprecipitation in OMV-treated VFFs will test association of exogenous bsRNAs with host AGO2. Two independent siRNAs targeting the endogenous AGO2 3′UTR will test whether Smad7 silencing and reporter effects depend on AGO2. Rescue with siRNA-resistant AGO2-WT or catalytically inactive AGO2-D669A will distinguish dependence on the AGO2 protein from dependence on its slicer activity, while wild-type and mutant Smad7 3′UTR dual-luciferase reporters will test target-site specificity; the work of Koeppen et al. has provided preliminary evidence for OMV-sRNA-mediated gene silencing within host cells, but their study design did not include AGO2-RIP, AGO2-dependency experiments, or 3′UTR reporter assays (15). Fifth, assessment of downstream fibrotic markers (phospho-Smad3, COL1A1, COL3A1, α-SMA) and rescue experiments with Smad7 overexpression or antagomir-mediated neutralization of the candidate bsRNA can confirm pathway specificity. Finally, fluorescent labeling for *in vivo* tracking of OMVs—a technique already applied to track OMV-RNA crossing the blood–brain barrier in a mouse model (31)—can be transferred to vocal fold mucosal barrier models, and integration into the dynamic vocal fold bioreactor system (59) would recapitulate the complete pathogenic cascade under physiologically relevant mechanical conditions. The existing research basis on the vocal fold epithelial barrier (19) and on the direct regulation of mucosal integrity by the laryngeal microbiota (24) can serve as entry points for *in vivo* validation. It should be acknowledged that the present hypothesis currently lacks direct experimental validation in vocal fold tissue and that some of the supporting evidence is extrapolated from other organ systems and experimental models; the validation pathway outlined above is intended to address these limitations systematically. A detailed experimental protocol specifying bacterial strains, culture conditions, OMV isolation procedures, co-culture parameters, controls, detection methods, and acceptance criteria for each validation step is provided in **Supplementary Protocol S1**.

**Figure 5:**
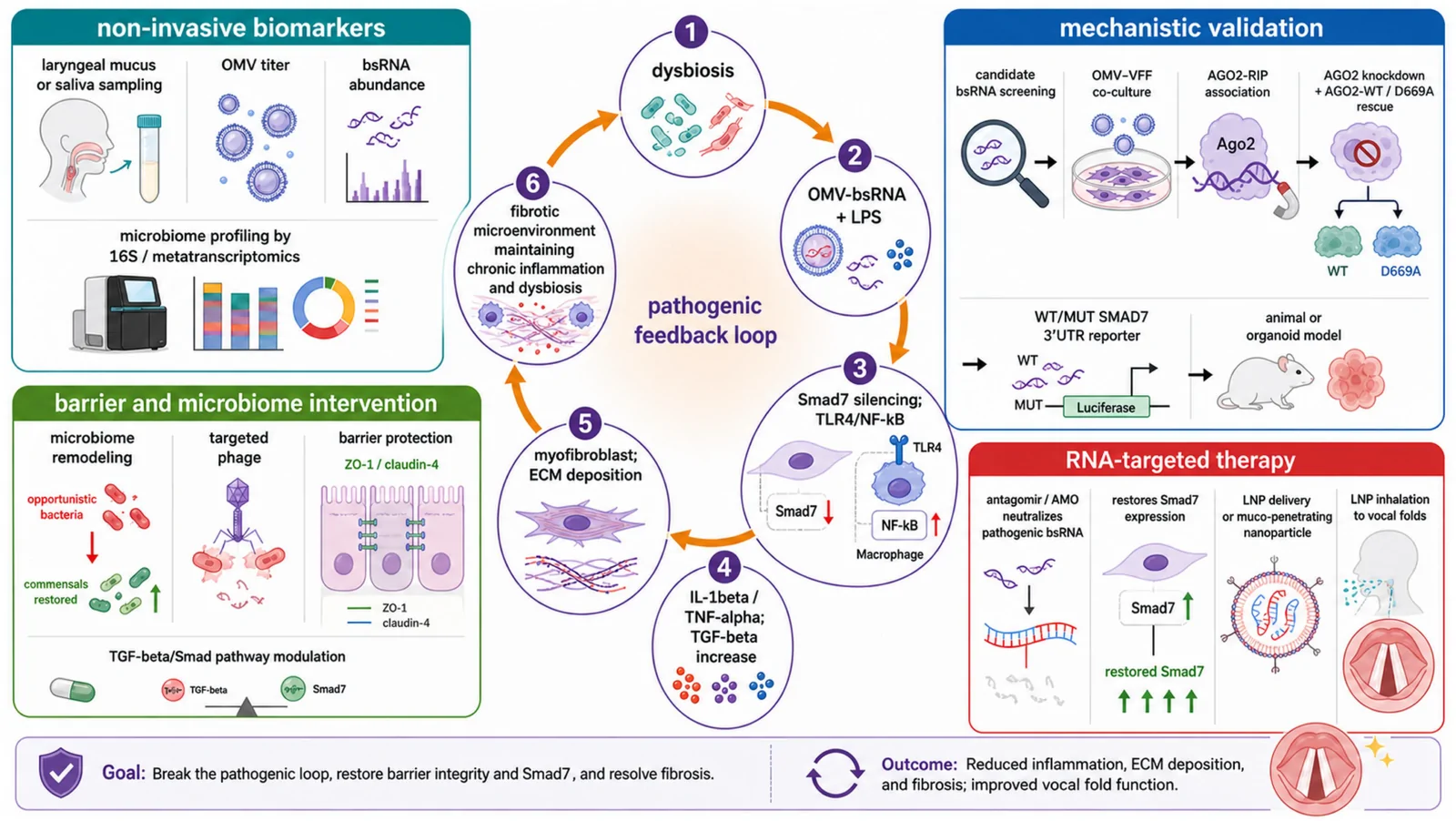
Validation pathway and intervention targets for vocal fold fibrosis based on the cross-kingdom RNA interference hypothesis.

### 5.3 Multi-target intervention strategies for vocal fold fibrosis

At the level of anti–vocal fold fibrosis therapy, and on the basis of the cross-kingdom interaction network, the following three directions hold translational promise.

#### 5.3.1 Laryngeal-targeted microbiota-remodeling therapy

Moving away from a treatment model of simply suppressing inflammation or overusing antibiotics, a “microbiota-remodeling aerosol” containing specific beneficial commensal strains or targeted bacteriophages could be designed for early intervention after phonotrauma, depriving opportunistic pathogens of their colonization advantage through competition for living space and nutrients and thereby reducing the release of pathogenic OMVs at the source (9). The prevention of scar formation by aerosol inhalation of phlorotannins in a laser-induced vocal fold fibrosis model demonstrates the feasibility of inhaled local drug-delivery strategies in vocal fold disease (60). A review of drug-delivery systems for wound healing in upper airway injury also provides a technical reference for the formulation design of this strategy (61).

#### 5.3.2 Inhaled nanoscale neutralizing agents based on RNA interference

For the identified pro-fibrotic pathogenic bsRNAs, antisense technology could be used to design high-affinity nucleic-acid antagonist sequences (antagomirs/antisense oligonucleotides, AMOs). Loading these antisense sequences into lipid nanoparticles capable of efficiently penetrating the mucosa for local administration would allow the specific neutralization and clearance of invading pathogenic bsRNAs within vocal fold cells, restoring the host’s intrinsic Smad7 gene expression and rebuilding Smad7-mediated negative-feedback regulation (62, 63). Lipid nanoparticles, as the principal carrier for nucleic-acid delivery, have been validated in the field of mRNA vaccines (64), and progress has also been made in the pulmonary delivery of lipid nanoparticle (LNP)-mRNA aerosolized by vibrating-mesh nebulizers (65). The application of inhaled nucleic-acid delivery systems in the treatment of pulmonary interstitial fibrosis indicates a technical transfer pathway for local RNA-targeted therapy of vocal fold fibrosis (66). The case of a Hic-5 antisense oligonucleotide inhibiting advanced hepatic fibrosis *in vivo* further corroborates the therapeutic efficacy of antisense-oligonucleotide strategies in parenchymal fibrotic organs (67). Epigenetic intervention strategies targeting DNA methylation and non-coding RNAs have shown promise in the treatment of cutaneous fibrosis and provide a reference for intervention at the epigenetic level in vocal fold fibrosis (68).

#### 5.3.3 Epithelial-barrier stress buffering and tight-junction reinforcement technology

Physicochemical protective preparations or vocal fold filler materials that maintain the structural stability of the epithelial tight junctions (ZO-1/claudin-4) could be developed to enhance the stress tolerance of the microscopic tissue scaffold during high-intensity, high-frequency physical collision and to reduce the risk of transbarrier infiltration of microbe-derived components (18, 20). Pirfenidone, an approved anti-fibrotic drug, has already shown anti-fibrotic activity in an *in vitro* model of vocal fold scarring, indicating that improved local delivery of existing anti-fibrotic drugs likewise holds translational value (69). The finding that tamoxifen inhibits vocal fold fibrosis by altering the TGF-β1/Smad signaling pathway also suggests a lead compound for the development of Smad-pathway-targeting drugs (70). A review of systematic strategies for targeting TGF-β signal transduction in fibrosis therapy can provide a reference for the selection of intervention targets (71).

This figure integrates the hypothesis-validation and translational-intervention pathways. The central loop shows the mutually reinforcing relationships among laryngeal dysbiosis, the sustained release of OMV-bsRNA and LPS, Smad7 silencing, TLR4/NF-κB activation, the increase in inflammatory cytokines and TGF-β, myofibroblast conversion, and ECM deposition. The peripheral modules comprise four categories of application: first, exploring noninvasive biomarkers by detecting OMV titer, bsRNA abundance, and microbial profiles in laryngeal surface mucus or saliva; second, validating the molecular mechanism through candidate bsRNA screening, OMV–vocal fold fibroblast co-culture, Ago2/RISC loading assays, and Smad7 3’UTR reporter-gene experiments; third, neutralizing pathogenic bsRNAs with antagomirs or AMOs and achieving local delivery via lipid nanoparticles or mucosa-penetrating nanoparticles; fourth, reducing fibrotic progression through microbiota remodeling, targeted bacteriophages, ZO-1/claudin-4 stabilization, and modulation of the TGF-β/Smad pathway.

## 6 Conclusion

This review introduces the mechanism of cross-kingdom RNA interference into the etiological framework of vocal fold fibrosis and proposes a pathogenic hypothesis that integrates mechanobiology, laryngeal microbiomics, and host RNA-interference mechanisms. High-frequency phonotrauma induces disassembly of the vocal fold epithelial tight junctions and laryngeal dysbiosis; the outer membrane vesicles released by opportunistic pathogens carry bacterial small RNAs across the damaged mucosal barrier to infiltrate the lamina propria, silence the host anti-fibrotic negative regulator Smad7 through a cross-kingdom route, and simultaneously activate the TLR4/NF-κB pathway of resident macrophages to release pro-inflammatory cytokines, forming a positive-feedback loop in which RNA interference and immune inflammation reinforce each other and ultimately driving tissue repair into an irreversible terminal fibrotic state. This hypothesis offers a mechanistic explanation for vocal fold fibrotic progression maintained by persistent laryngeal dysbiosis after the initiating mechanical injury has resolved, and provides a theoretical basis for anti-fibrotic strategies based on laryngeal microbiota modulation and RNA-targeted delivery. Although the proposed pathogenic network remains hypothetical and awaits experimental validation, further dissection and testing of this model may advance the renewal of diagnostic and therapeutic strategies for vocal fold scarring.
