# Supplementary material for: Cross-kingdom RNA interference in the dysbiosis-mediated network of vocal fold fibrosis: a pathogenic hypothesis and molecular intervention targets
Source: Front Immunol. 2026 Sep 15;17:1923591. doi: 10.3389/fimmu.2026.1923591 (PMC13620465; doi:10.3389/fimmu.2026.1923591)
Supplement: Supplementary file 1 [file DataSheet1.docx]

**Supplementary Protocol S1: Proposed In Vitro Experimental System for Validating the Cross-Kingdom RNA Interference Hypothesis in Vocal Fold Fibrosis**

## Overview

This supplementary protocol describes a five-step in vitro experimental system designed to test the central hypothesis that bacterial small RNAs (bsRNAs) in outer membrane vesicles (OMVs) from laryngeal opportunistic pathogens cross the mucosal barrier, enter vocal fold fibroblasts (VFFs), load onto host AGO2/RISC, and post-transcriptionally silence *Smad7*, thereby releasing TGF-beta/Smad3 pro-fibrotic signaling and driving vocal fold fibrosis. The five steps are organized in a sequential framework:

- **Step 1 (Candidate Screening and Ranking)** identifies bsRNAs complementary to the human *Smad7* 3′UTR and ranks them by reproducibility of RNA termini and dominant length, 5′ chemistry, structure, OMV enrichment, and AGO2-loading compatibility.

- **Step 2 (OMV Isolation and Characterization)** obtains and validates OMVs from laryngeal opportunistic pathogens, confirming the presence and identity of bsRNA cargo.

- **Step 3 (OMV-VFF Co-culture)** tests whether OMV exposure reduces Smad7 expression in VFFs in a dose- and time-dependent manner.

- **Step 4 (AGO2 Association, Dependency, and Target Specificity)** combines AGO2-RIP, wild-type/mutant *Smad7* 3′UTR reporters, AGO2 knockdown, and AGO2-WT/D669A rescue to distinguish association, target-site specificity, functional dependence, and slicer activity.

- **Step 5 (Fibrotic Phenotype Assessment and Rescue)** assesses whether Smad7 silencing leads to downstream pro-fibrotic changes and whether these changes are reversible by Smad7 overexpression or bsRNA neutralization.

## Step 1: Bioinformatic Screening of Candidate bsRNAs Targeting the Smad7 3'UTR

### Rationale

This step screens the sRNA repertoire of laryngeal pathogen OMVs for candidate bsRNAs with sequence complementarity to the human *Smad7* 3'UTR. Koeppen et al. demonstrated that sRNA52320 in *P. aeruginosa* OMVs targets human MAP2K1/MAP2K2 mRNAs through partial sequence complementarity [S1]; this step applies the same bioinformatic strategy to Smad7.

### Procedure

1. **Retrieve the target sequence.** Download the full-length 3'UTR of human *Smad7* mRNA (1,847 nt; NCBI RefSeq NM_005904.4) from the NCBI Nucleotide database.

2. **Compile the bsRNA query set.** Obtain the annotated sRNA repertoire of *Pseudomonas aeruginosa* PAO1 and *Fusobacterium nucleatum* ATCC 25586 from the following databases:

- Bacterial Small Regulatory RNA Database (BSRD; http://www.bsrd.org)

- sRNAmap (http://srnamap.mbc.nctu.edu.tw)

- Rfam (https://rfam.org)

Additionally, include OMV-enriched RNAs identified by small RNA sequencing and classify them as independently transcribed regulatory sRNAs, RNase E/RNase III-processed short RNAs, tRNA-derived fragments, rRNA-derived fragments, or short RNAs of undetermined origin. Very small RNAs (15–30 nt) may enter the initial query set, but their 5′ ends, 3′ ends, and dominant lengths must be reproducible across independent biological replicates, and stable OMV enrichment must be demonstrated; RNA origin or length alone does not establish regulatory function [S6].

3. **Predict RNA-RNA interactions using IntaRNA 2.0.** Submit pairwise queries (each bsRNA against the *Smad7* 3'UTR) to IntaRNA 2.0 (http://rna.informatik.uni-freiburg.de/IntaRNA/) [S2]. Use the following parameters:

- Minimum seed length: 7 nt continuous complementarity

- Energy model: Turner 2004

- Accessibility computation: enabled (RNAplfold-based, window size = 150 nt, max base-pair span = 100 nt)

- Output: top 5 interaction sites per query, ranked by minimum free energy (MFE)

For detailed parameter optimization, follow the use-case-driven handbook for IntaRNA [S3].

4. **Apply filtering criteria.** First retain candidates that meet the targeting thresholds below, and then assess biochemical compatibility with host AGO2 loading [S27, S29]:

- Predicted MFE <= -20 kcal/mol

- Seed-region complementarity >= 7 nt continuous match in the 5' end of the bsRNA (positions 1-8, analogous to miRNA seed regions)

- Accessibility of the target site on *Smad7* 3'UTR (ED value < 10 kcal/mol, indicating the binding region is not occluded by secondary structure)

- The predominant 5′ end, 3′ end, and dominant length are consistent across at least three independent biological replicates

- Prioritize candidates of approximately 21–23 nt with a 5′U or 5′A; retain candidates of a similar length or with 5′C/G as secondary-priority candidates when stable OMV enrichment and host-functional evidence are present

- Determine whether the native 5′ end is monophosphorylated by 5′-phosphate-dependent adaptor ligation, with comparison before and after RNA 5′-pyrophosphohydrolase treatment

- Use RNAfold to evaluate duplex regions and 3′-end structures of the candidate and its putative precursor; a canonical 2-nt 3′ overhang is a priority feature rather than an exclusion criterion

- The candidate shows higher normalized abundance in the OMV fraction than in whole-cell bacterial RNA and is reproducibly detected across independent OMV preparations

5. **Cross-validate with SPOT.** Submit the top candidates to the sRNA Target Prediction Organizing Tool (SPOT) [S4] to integrate computational predictions with available experimental evidence from bacterial systems and to rank candidates by a composite confidence score.

6. **Assess conservation.** For each candidate bsRNA, perform BLAST searches against the genomes of related laryngeal opportunistic pathogens (e.g., *P. aeruginosa* clinical isolates, *F. nucleatum* subspecies) to evaluate the conservation of the predicted targeting sequence across strains.

7. **Rank candidates.** Class A candidates meet all targeting thresholds, show reproducible 5′/3′ ends and dominant lengths, carry a native 5′ monophosphate, preferentially begin with 5′U/A, possess an AGO-compatible structure, and are stably enriched in OMVs. Class B candidates meet the targeting thresholds and show reproducible termini, dominant lengths, and stable OMV enrichment, but have a noncanonical 5′ nucleotide, length, or duplex-end structure. Both classes proceed to AGO2-RIP and functional testing, with Class A prioritized.

### Acceptance criteria

Candidate bsRNAs must meet MFE <= -20 kcal/mol, >= 7 nt seed-region complementarity to the *Smad7* 3'UTR, and target-site ED < 10 kcal/mol. Priority is then assigned according to reproducibility of 5′ ends, 3′ ends, and dominant lengths; 5′-phosphorylation status; the 5′ nucleotide; duplex and 3′-end structure; and stable OMV enrichment. Class A and Class B candidates proceed to AGO2-loading, AGO2-dependency, and *Smad7* 3′UTR target-specificity testing.

## Step 2: OMV Isolation and Characterization

### Rationale

This step isolates OMVs from laryngeal opportunistic pathogens, characterizes them per MISEV2023 guidelines [S9], and profiles their bsRNA cargo by small RNA sequencing.

### Materials and bacterial strains

| **Item** | **Specification** |
| --- | --- |
| *Pseudomonas aeruginosa* | Strain PAO1 (ATCC 15692) |
| *Fusobacterium nucleatum* | Strain ATCC 25586 (subsp. nucleatum) |
| Growth medium (*P. aeruginosa*) | Luria-Bertani (LB) broth or minimal medium M9 supplemented with 0.4% glucose |
| Growth medium (*F. nucleatum*) | Brain Heart Infusion (BHI) broth supplemented with hemin (5 microg/mL) and menadione (1 microg/mL) |
| Anaerobic chamber | For *F. nucleatum* culture (85% N2, 10% H2, 5% CO2) |
| Ultracentrifuge | Ultracentrifuge with appropriate rotor |
| Nanoparticle tracking analysis (NTA) | NTA instrument |
| Transmission electron microscope (TEM) | TEM with negative staining capability |
| Small RNA sequencing platform | high-throughput sequencing platform |

### Procedure

**2.1 Bacterial culture and stress induction**

1. **P. aeruginosa PAO1:** Inoculate a single colony into 10 mL LB broth and grow overnight at 37 degrees C with shaking (200 rpm). Subculture at 1:100 dilution into 500 mL LB broth. To simulate dysbiotic stress conditions, supplement with sub-inhibitory concentrations of gentamicin (0.5 microg/mL, 1/4 MIC) or grow under iron-limited conditions (addition of 200 microM 2,2'-dipyridyl) [S1]. Harvest culture at early stationary phase (OD600 = 1.8-2.0, approximately 16-18 h).

2. **F. nucleatum ATCC 25586:** Inoculate into 10 mL BHI broth supplemented with hemin/menadione and grow anaerobically at 37 degrees C for 48 h. Subculture at 1:50 into 500 mL supplemented BHI. To simulate stress, grow under oxidative stress (addition of 0.25 mM H2O2 at mid-log phase) or at mildly acidic pH (pH 6.5, adjusted with HCl) [S10, S11]. Harvest at early stationary phase (OD600 = 0.8-1.0, approximately 48-72 h).

**2.2 OMV isolation by density-gradient ultracentrifugation**

Perform all steps at 4 degrees C unless otherwise specified. Adapt the protocol from Melo et al. [S8]:

1. Remove bacteria by centrifugation at 6,000 x g for 20 min.

2. Filter the supernatant through a 0.45-micrometer polyethersulfone (PES) membrane, followed by a 0.22-micrometer PES membrane, to remove residual cells and large debris.

3. Concentrate the filtered supernatant by ultrafiltration using a 100 kDa molecular weight cutoff (MWCO) hollow-fiber cartridge or 100 kDa MWCO centrifugal filter units.

4. Pellet crude OMVs by ultracentrifugation at 150,000 x g for 3 h at 4 degrees C (SW 32 Ti rotor).

5. Resuspend the pellet in 1 mL sterile phosphate-buffered saline (PBS, pH 7.4).

6. Overlay onto a discontinuous OptiPrep (iodixanol) density gradient (10%, 20%, 30%, 40%, 50% w/v in PBS) and centrifuge at 100,000 x g for 18 h at 4 degrees C (SW 41 Ti rotor).

7. Collect 1-mL fractions from the top. OMV-containing fractions typically sediment at densities of 1.13-1.19 g/mL (fractions corresponding to the 20-30% iodixanol interface).

8. Pool OMV-positive fractions, dilute with PBS, and pellet by ultracentrifugation at 150,000 x g for 3 h.

9. Resuspend purified OMVs in sterile PBS and store at -80 degrees C in single-use aliquots. Avoid repeated freeze-thaw cycles.

**2.3 OMV characterization**

1. **Protein quantification:** Determine OMV protein concentration by bicinchoninic acid (BCA) assay . Express OMV dosing in subsequent experiments as microg protein/mL.

2. **Nanoparticle tracking analysis (NTA):** Dilute OMV samples 1:100-1:1000 in particle-free PBS. Acquire 5 x 60-s videos per sample at camera level 13-15 . Report modal particle diameter and concentration (particles/mL). Expected size distribution: 30-200 nm with a modal diameter of 50-100 nm.

3. **Transmission electron microscopy (TEM):** Adsorb 5 microL OMV suspension onto carbon-coated 300-mesh copper grids for 2 min. Negatively stain with 2% uranyl acetate for 1 min. Image at 80-100 kV. Confirm spherical, bilayered morphology.

4. **Western blot for OMV markers:** Probe for positive markers (OmpA or OmpC for *P. aeruginosa*; FomA for *F. nucleatum*) and negative markers (cytoplasmic markers such as GroEL or RecA, to confirm absence of bacterial cell contamination), in accordance with MISEV2023 recommendations [S9].

5. **Sterility verification:** Plate 100 microL OMV suspension on LB agar (for *P. aeruginosa*) or BHI agar (for *F. nucleatum*) and incubate for 48-72 h to confirm the absence of viable bacteria.

**2.4 Small RNA extraction and sequencing**

1. Treat purified OMVs with RNase A (10 microg/mL, 37 degrees C, 30 min) followed by proteinase K (50 microg/mL, 37 degrees C, 30 min) to remove surface-associated RNA and protein, then wash with PBS by ultracentrifugation (150,000 x g, 1 h).

2. Extract total RNA from RNase-treated OMVs using the miRNeasy Mini Kit or TRIzol LS according to the manufacturer's protocol.

3. Assess RNA quality and size distribution using a Bioanalyzer with the Small RNA chip.

4. Construct small RNA libraries using the NEBNext Small RNA Library Prep Set or equivalent.

5. Sequence on the Illumina NovaSeq 6000 platform (single-end 50 bp, minimum 10 million reads per sample).

6. Analyze reads: adapter trimming (Cutadapt), quality filtering (Phred >= 30), alignment to the respective bacterial genome (Bowtie2), and annotation against Rfam, BSRD, and sRNAmap databases. Reads mapping to neither rRNA nor tRNA and falling within 15-50 nt are classified as candidate regulatory sRNAs [S5, S6].

### Quality control criteria (MISEV2023 compliance)

In accordance with MISEV2023 [S9], the following criteria must be met before OMVs are used in downstream experiments:

| **Parameter** | **Acceptance criterion** |
| --- | --- |
| Modal diameter (NTA) | 30-200 nm |
| Morphology (TEM) | Spherical, intact bilayer membrane |
| Positive protein markers | Detection of >= 1 transmembrane protein and >= 1 lipid-bound protein |
| Negative markers | Absence of cytoplasmic markers (GroEL, RecA) |
| Sterility | No colony growth after 72 h incubation |
| Endotoxin (for RNA-specific controls) | Quantified by Limulus amebocyte lysate (LAL) assay |
| RNA yield | Detectable small RNA peak (15-50 nt) on Bioanalyzer |

## Step 3: OMV-VFF Co-culture and Smad7 Expression Analysis

### Rationale

This step tests whether OMVs can be taken up by VFFs and whether OMV exposure leads to a reduction in Smad7 mRNA and protein levels.

### Cell source and culture

1. **Cell line:** Use the SV40-immortalized human vocal fold fibroblast cell line established by Chu et al. [S12], which retains fibroblast morphology and functional markers and provides standardization and reproducibility. Culture in Dulbecco's Modified Eagle Medium (DMEM) supplemented with 10% fetal bovine serum (FBS), 100 U/mL penicillin, 100 microg/mL streptomycin, and 2 mM L-glutamine at 37 degrees C in 5% CO2 [S13, S14].

2. **Cell seeding:** Seed VFFs at 2 x 10^5 cells per well in 6-well plates (for RNA/protein extraction) or at 5 x 10^4 cells per well on glass coverslips in 24-well plates (for fluorescence microscopy). Allow cells to reach 60-70% confluence before OMV treatment. Serum-starve cells in DMEM with 0.5% FBS for 12 h before OMV addition to reduce baseline TGF-beta signaling [S14].

### OMV dosing and time course

Treat VFFs with purified OMVs from *P. aeruginosa* PAO1 or *F. nucleatum* ATCC 25586 at the following concentrations and time points:

| **Parameter** | **Values** |
| --- | --- |
| OMV concentration (protein equivalent) | 1, 10, 50 microg/mL |
| Co-culture duration | 6, 12, 24, 48 h |
| Biological replicates per condition | n >= 3 |
| Technical replicates per biological replicate | n >= 2 (for RT-qPCR) |

The concentration range of 1-50 microg/mL protein equivalent is based on established OMV co-culture protocols in mammalian cells [S1, S10, S17].

### Controls

| **Control** | **Purpose** |
| --- | --- |
| Untreated VFFs (PBS vehicle) | Baseline Smad7 expression |
| Heat-inactivated OMVs (95 degrees C, 15 min) | Denature RNA cargo while preserving LPS; distinguish RNA-mediated effects from LPS-driven responses |
| RNase A-pretreated OMVs (10 microg/mL RNase A, 37 degrees C, 30 min, followed by RNase inhibitor addition) | Degrade surface-associated RNA; assess contribution of externally associated versus internally packaged RNA |
| Proteinase K-pretreated OMVs (50 microg/mL, 37 degrees C, 30 min, followed by PMSF inactivation) | Degrade surface proteins involved in OMV uptake; assess protein-dependent uptake mechanisms |
| TGF-beta1 positive control (10 ng/mL recombinant human TGF-beta1) | Confirm VFF responsiveness to pro-fibrotic stimulation and Smad pathway competence [S14] |

### OMV uptake verification

1. **Fluorescent lipid labeling:** Label purified OMVs with PKH26 Red Fluorescent Cell Linker according to the manufacturer's protocol. Remove unincorporated dye by washing with PBS through 100 kDa ultrafiltration units (3 washes).

2. **Co-culture and imaging:** Incubate PKH26-labeled OMVs with VFFs at 10 microg/mL for 2, 6, and 12 h. Fix cells with 4% paraformaldehyde (15 min, room temperature), stain nuclei with DAPI (1 microg/mL, 5 min), and mount.

3. **Confocal microscopy:** Image using a confocal laser scanning microscope with 63x oil-immersion objective. Acquire z-stack images (0.5 micrometer intervals) to confirm intracellular localization of PKH26 signal. Perform orthogonal cross-sectional analysis to distinguish internalized OMVs from surface-bound particles [S15, S16].

4. **Flow cytometry quantification:** Trypsinize and wash cells after OMV treatment to remove surface-bound OMVs. Quantify the percentage of PKH26-positive cells and mean fluorescence intensity (MFI) by flow cytometry. Include an unlabeled OMV control to set the gating threshold.

### Detection methods

**RT-qPCR for Smad7 mRNA**

1. Extract total RNA using TRIzol试剂 according to the manufacturer's protocol.

2. Treat with DNase I to remove genomic DNA contamination.

3. Reverse-transcribe 1 microg total RNA using the PrimeScript RT试剂盒 with oligo(dT) and random hexamer primers.

4. Perform quantitative PCR using TB Green Premix Ex Taq II on a real-time qPCR instrument system.

5. Primers:

- *SMAD7* forward: 5'-CCTTAGCCGACTCTGCGAACTA-3'

- *SMAD7* reverse: 5'-CCAGATAATTCGTTCCCCCTGT-3'

- *GAPDH* forward: 5'-GCACCGTCAAGGCTGAGAAC-3'

- *GAPDH* reverse: 5'-TGGTGAAGACGCCAGTGGA-3'

6. Cycling conditions: 95 degrees C for 30 s; 40 cycles of 95 degrees C for 5 s, 60 degrees C for 30 s; followed by melting curve analysis.

7. Use GAPDH as the internal reference to calibrate Smad7 expression by the 2^(-Delta Delta Ct) method. Additionally validate with a second reference gene (e.g., *ACTB* or *RPL13A*).

**Western blot for Smad7 protein**

1. Lyse cells in RIPA buffer supplemented with protease inhibitor cocktail and phosphatase inhibitor cocktail on ice for 30 min.

2. Quantify protein by BCA assay.

3. Load 30 microg total protein per lane on 10% SDS-PAGE gels.

4. Transfer to PVDF membrane (0.45 micrometer) at 100 V for 90 min in cold transfer buffer.

5. Block with 5% non-fat milk in TBS-T (0.1% Tween-20) for 1 h at room temperature.

6. Primary antibodies (4 degrees C, overnight):

- Anti-Smad7 (1:1000)

- Anti-GAPDH (1:5000) as loading control

7. Secondary antibody: HRP-conjugated goat anti-mouse or anti-rabbit IgG (1:5000; 1 h, room temperature).

8. Detect by enhanced chemiluminescence (ECL). Quantify band intensity using ImageJ or Image Lab software. Use GAPDH as the internal reference to calibrate Smad7 protein levels.

### Acceptance criteria

Use a ≥30% reduction in Smad7 protein (Western blot densitometry, p < 0.05) as the primary acceptance criterion; Smad7 mRNA reduction measured by RT-qPCR serves as a supportive endpoint. Compare native OMVs versus heat-inactivated OMVs and RNase-pretreated OMVs to determine whether the observed effect is RNA-dependent.

## Step 4: AGO2-RIP and Dual-Luciferase Reporter Assay

### Rationale

AGO2-RIP will test whether candidate bsRNAs form complexes with host AGO2 [S18, S19], wild-type/mutant *Smad7* 3′UTR dual-luciferase assays will test target-site specificity [S22, S23], and AGO2 knockdown followed by rescue with AGO2-WT or catalytically inactive AGO2-D669A will determine functional dependence and the contribution of slicer activity [S28].

### AGO2-RIP procedure

Perform AGO2-RIP following established protocols [S18] with modifications for detecting exogenous bacterial sRNAs:

1. **Cell treatment:** Seed VFFs in 150-mm dishes (5 x 10^6 cells per dish; 3 dishes per condition). Treat with purified OMVs (10 microg/mL) from *P. aeruginosa* PAO1 or *F. nucleatum* ATCC 25586 for 24 h. Include an untreated control dish.

2. **Cross-linking (optional, for enhanced recovery):** UV cross-link cells at 254 nm (150 mJ/cm^2) using a Stratalinker 2400, to stabilize RNA-protein interactions in situ.

3. **Cell lysis:** Wash cells twice with ice-cold PBS. Lyse in polysome lysis buffer (100 mM KCl, 5 mM MgCl2, 10 mM HEPES pH 7.0, 0.5% NP-40, 1 mM DTT, 100 U/mL RNase inhibitor , 1x protease inhibitor cocktail). Incubate on ice for 10 min, then centrifuge at 14,000 x g for 15 min at 4 degrees C. Reserve 10% of the lysate as input.

4. **Immunoprecipitation:** Pre-coat Protein G magnetic beads (50 microL per reaction) with 5 microg anti-AGO2 antibody (clone 11A9) or 5 microg mouse IgG isotype control (negative control) by incubation at 4 degrees C for 4 h with rotation. Add pre-cleared lysate to antibody-coated beads and incubate overnight at 4 degrees C with gentle rotation.

5. **Washes:** Wash beads 4 times with NT2 buffer (50 mM Tris-HCl pH 7.4, 150 mM NaCl, 1 mM MgCl2, 0.05% NP-40, 1 mM DTT, 20 mM EDTA) for 5 min each at 4 degrees C.

6. **RNA extraction:** Elute RNA from washed beads by adding 700 microL QIAzol directly to the beads. Extract RNA using the miRNeasy Mini Kit according to the manufacturer's instructions.

7. **Detection of exogenous bsRNAs in the AGO2-RIP eluate:**

- **RT-qPCR with species-specific primers:** Design primers targeting the candidate bsRNA that anneal to sequences unique to the bacterial genome and absent from the human genome. As a positive control for AGO2-RIP efficiency, detect endogenous miR-21 (a well-characterized AGO2-loaded human miRNA). As a negative control, detect U6 snRNA (not loaded onto AGO2).

- **Small RNA sequencing of RIP eluate:** Construct and sequence small RNA libraries from the AGO2-RIP eluate . Align reads against both the human genome (hg38) and the respective bacterial genome. The enrichment of candidate bsRNAs in the AGO2-RIP fraction relative to the IgG control fraction (fold enrichment >= 5, determined by calibrated read counts) constitutes evidence of RISC loading [S1, S19].

8. **Western blot verification:** Confirm successful AGO2 immunoprecipitation by Western blot of the bead eluate using anti-AGO2 antibody.

### Reporter construct design

Design and clone dual-luciferase reporter constructs following established methods [S22, S23, S24]:

1. **Wild-type construct (pGL3-Smad7-3'UTR-WT):** Amplify a 200-400 bp fragment of the *Smad7* 3'UTR encompassing the predicted bsRNA binding site(s) by PCR from human genomic DNA. Clone the fragment downstream of the firefly luciferase (*luc2*) coding sequence in the pmirGLO Dual-Luciferase miRNA Target Expression Vector at the XhoI/NotI restriction sites. The vector co-expresses *Renilla* luciferase (hRluc-neo) from an independent promoter to correct for transfection efficiency differences.

2. **Mutant construct (pGL3-Smad7-3'UTR-MUT):** Introduce 4-nucleotide substitutions (transversions) within the seed-complementary region of the predicted bsRNA binding site using site-directed mutagenesis . Verify mutations by Sanger sequencing. This mutant control distinguishes sequence-specific targeting from non-specific effects on the reporter [S22, S24].

3. **bsRNA mimic:** Synthesize the candidate bsRNA as a chemically modified double-stranded RNA oligonucleotide (2'-O-methyl modifications on the passenger strand to ensure preferential guide-strand loading), at a purity of >= 95% (HPLC-purified).

4. **Scrambled control oligonucleotide:** Synthesize a scrambled-sequence RNA oligonucleotide of the same length and GC content as the bsRNA mimic, with no predicted targets in the human transcriptome (verified by BLAST).

**AGO2 knockdown and rescue**

1. **AGO2 knockdown:** Transfect VFFs with two independent siRNAs targeting different sequences in the 3′UTR of endogenous *AGO2* mRNA and with a non-targeting siRNA control. At 48 h, confirm >= 70% reduction of AGO2 protein by Western blot and assess cell viability and AGO1, AGO3, and AGO4 expression.

2. **Treatment conditions:** Add native OMVs or transfect the bsRNA mimic in AGO2-depleted and control cells; analyze native-OMV and mimic experiments separately.

3. **Rescue constructs:** In AGO2-depleted cells, express an siRNA-resistant AGO2-WT construct lacking the targeted endogenous 3′UTR, catalytically inactive AGO2-D669A, or empty vector. Confirm comparable AGO2-WT and AGO2-D669A expression by Western blot.

4. **Primary readouts:** Measure Smad7 protein, wild-type/mutant *Smad7* 3′UTR reporter activity, and p-Smad3; Smad7 mRNA serves as a supportive endpoint.

5. **Interpretation:** Attenuation after AGO2 knockdown with restoration by AGO2-WT establishes AGO2 functional dependence. Rescue by AGO2-D669A supports non-slicing post-transcriptional silencing; rescue only by AGO2-WT indicates involvement of AGO2 catalytic activity [S28].

### Positive and negative controls

| **Control** | **Purpose** |
| --- | --- |
| bsRNA mimic + pGL3-Smad7-3'UTR-WT | Test for sequence-specific silencing |
| bsRNA mimic + pGL3-Smad7-3'UTR-MUT | Confirm seed-region specificity |
| Scrambled oligo + pGL3-Smad7-3'UTR-WT | Exclude non-specific RNA effects |
| Untransfected cells + pGL3-Smad7-3'UTR-WT | Baseline luciferase activity |
| miR-21 mimic + pGL3-PDCD4-3'UTR (positive control vector) | Validate the reporter assay system [S22] |
| IgG isotype control (for AGO2-RIP) | Non-specific immunoprecipitation background |
| U6 snRNA detection (for AGO2-RIP) | Negative control for AGO2 loading specificity |
| Non-targeting siRNA + OMV/bsRNA mimic | AGO2-knockdown negative control |
| AGO2 siRNA-1 or siRNA-2 + OMV/bsRNA mimic | Test AGO2 functional dependence |
| AGO2 siRNA + empty vector | Rescue baseline control |
| AGO2 siRNA + siRNA-resistant AGO2-WT | Rescue AGO2 functional dependence |
| AGO2 siRNA + siRNA-resistant AGO2-D669A | Distinguish AGO2-protein dependence from slicer-activity dependence |

### Dual-luciferase reporter assay procedure

1. Seed HEK-293T cells (for high transfection efficiency) or VFFs (for tissue relevance) at 1 x 10^4 cells per well in white opaque 96-well plates, 24 h before transfection.

2. Co-transfect 100 ng reporter construct (WT or MUT) with 50 nM bsRNA mimic or scrambled control using Lipofectamine 3000 according to the manufacturer's protocol. Each condition in triplicate.

3. At 48 h post-transfection, assay firefly and *Renilla* luciferase activities sequentially using the Dual-Luciferase Reporter Assay System on a microplate luminometer.

4. Calculate the firefly/*Renilla* ratio for each well. Use the scrambled control group as the baseline to calculate relative values, correcting for non-specific RNA effects.

### Acceptance criteria

- **AGO2-RIP positive result:** Candidate bsRNAs are enriched >= 5-fold in the AGO2-RIP eluate compared to the IgG control in OMV-treated VFFs but not in untreated VFFs, supporting association of exogenous bsRNAs with host AGO2 after OMV uptake.

- **Reporter assay positive result:** The bsRNA mimic produces a >= 50% reduction in firefly/*Renilla* luciferase ratio for the wild-type *Smad7* 3'UTR construct but not for the mutant construct, and the scrambled control does not reduce luciferase activity for either construct. This pattern demonstrates sequence-specific, seed-region-dependent targeting of *Smad7* [S22].

- **AGO2 functional dependence:** Both independent AGO2 siRNAs should attenuate the native-OMV- or bsRNA-mimic-induced reduction in Smad7 protein, suppression of the wild-type 3′UTR reporter, and increase in p-Smad3; siRNA-resistant AGO2-WT should restore these effects.

- **Catalytic-activity assessment:** Rescue by AGO2-D669A supports AGO2-dependent but slicer-independent post-transcriptional silencing; rescue only by AGO2-WT indicates involvement of AGO2 catalytic activity. The mutant *Smad7* 3′UTR reporter should not show the same pattern.

## Step 5: Fibrotic Phenotype Assessment and Rescue Experiments

### Rationale

This step assesses downstream fibrotic phenotype changes (phospho-Smad3, collagen, α-SMA) in OMV-treated VFFs and tests pathway specificity through Smad7 overexpression and antagomir-mediated rescue experiments.

### Markers and detection

Assess the following fibrotic markers in OMV-treated VFFs at the optimal dose and time point (10-50 microg/mL, 24-48 h) [S25, S26]:

| **Marker** | **Method** | **Antibody/Primer** | **Expected change** |
| --- | --- | --- | --- |
| Smad7 protein | Western blot | Anti-Smad7 | Decreased |
| Phospho-Smad3 (Ser423/425) | Western blot | Anti-p-Smad3 | Increased |
| Total Smad3 | Western blot | Anti-Smad3 | Unchanged |
| COL1A1 mRNA | RT-qPCR | Forward: 5'-GAGGGCCAAGACGAAGACATC-3'; Reverse: 5'-CAGATCACGTCATCGCACAAC-3' | Increased (>2-fold) |
| COL3A1 mRNA | RT-qPCR | Forward: 5'-TTGAAGGAGGATGTTCCCATCT-3'; Reverse: 5'-ACAGACACATATTTGGCATGGTT-3' | Increased (>2-fold) |
| alpha-SMA (ACTA2) | Immunofluorescence + RT-qPCR | Anti-alpha-SMA (1:200) + primers (F: 5'-GTGTTGCCCCTGAAGAGCAT-3'; R: 5'-GCTGGGACATTGAAAGTCTCA-3') | Increased |
| Fibronectin (FN1) | Western blot | Anti-fibronectin (1:1000) | Increased |

**Immunofluorescence for alpha-SMA**

1. Fix VFFs on coverslips with 4% paraformaldehyde (15 min, room temperature).

2. Permeabilize with 0.1% Triton X-100 in PBS (10 min).

3. Block with 5% normal goat serum in PBS (1 h, room temperature).

4. Incubate with anti-alpha-SMA antibody (1:200) overnight at 4 degrees C.

5. Wash 3 times with PBS.

6. Incubate with Alexa Fluor 488-conjugated secondary antibody (1:500; 1 h, room temperature, protected from light).

7. Counterstain nuclei with DAPI (1 microg/mL, 5 min).

8. Mount and image by confocal microscopy. Quantify the percentage of alpha-SMA-positive cells and mean fluorescence intensity across >= 5 fields per condition.

**Collagen deposition assay (Sirius Red)**

1. At 72 h post-OMV treatment, fix VFF monolayers in Bouin's solution (1 h, room temperature).

2. Stain with Sirius Red (0.1% Direct Red 80 in saturated picric acid) for 1 h.

3. Wash with 0.01 N HCl.

4. Dissolve bound dye in 0.1 N NaOH and measure absorbance at 540 nm.

5. Correct by cell number (determined by crystal violet staining of parallel wells).

### Rescue approaches

**Rescue 1: Smad7 overexpression**

1. **Construct:** Clone the full-length human *SMAD7* coding sequence (NCBI RefSeq NM_005904.4) into the pcDNA3.1(+) expression vector or a lentiviral expression vector (e.g., pLVX-IRES-Puro). Include a GFP-tagged version to monitor transfection/transduction efficiency.

2. **Transfection:** Transfect VFFs with pcDNA3.1-Smad7 or pcDNA3.1-empty vector (control) using Lipofectamine 3000 at a ratio of 1:3 (DNA:reagent). At 24 h post-transfection, add OMVs (10 microg/mL) for an additional 24-48 h.

3. **Alternatively, lentiviral transduction:** Transduce VFFs with lentivirus encoding Smad7 or empty vector at MOI = 10. Select with puromycin (2 microg/mL) for 48 h, then treat with OMVs.

4. **Readout:** Assess the same fibrotic markers as above. If the hypothesis is correct, Smad7 overexpression should prevent or substantially attenuate the OMV-induced increase in phospho-Smad3, COL1A1, COL3A1, and alpha-SMA.

**Rescue 2: Antagomir-mediated bsRNA neutralization**

1. **Design:** Synthesize a 2'-O-methyl-modified, phosphorothioate-backbone antisense oligonucleotide (antagomir) fully complementary to the candidate bsRNA . Also synthesize a scrambled antagomir of the same length and modification pattern as a negative control.

2. **Transfection:** Transfect VFFs with 100 nM antagomir or scrambled control using Lipofectamine RNAiMAX 6 h before OMV addition.

3. **Readout:** If the fibrotic response is specifically mediated by the candidate bsRNA, the antagomir (but not the scrambled control) should prevent Smad7 downregulation and the downstream fibrotic phenotype.

**Rescue 3: SB431542 (TGF-beta type I receptor kinase inhibitor)**

1. Pre-treat VFFs with SB431542 (10 microM) for 1 h before OMV addition.

2. This pharmacological control tests whether the observed fibrotic effects are TGF-beta/Smad3-pathway-dependent, regardless of the mechanism of Smad7 downregulation.

### Acceptance criteria

| **Condition** | **Readout and assessment** |
| --- | --- |
| OMV-treated vs. untreated VFFs | Reduced Smad7 protein; increased p-Smad3, COL1A1 (>2-fold), COL3A1 (>2-fold), alpha-SMA |
| OMV + Smad7 overexpression | Attenuation (>= 50% reversal) of fibrotic markers |
| OMV + antagomir | Prevention of Smad7 downregulation and fibrotic phenotype |
| OMV + scrambled antagomir | No rescue (fibrotic phenotype persists) |
| Heat-inactivated OMV | Attenuated or absent fibrotic response |
| SB431542 + OMV | Blocked phospho-Smad3 and downstream fibrotic markers |

- **Complete confirmation:** OMVs induce pro-fibrotic changes that are (a) RNA-dependent (attenuated by heat inactivation), (b) bsRNA-specific (blocked by antagomir but not scrambled control), (c) Smad7-dependent (rescued by Smad7 overexpression), and (d) TGF-beta/Smad3-pathway-dependent (blocked by SB431542).

## Summary Table: Hypothesis Steps and Validation Approaches

| **Step** | **Hypothesis component** | **Validation approach** | **Key techniques** | **Acceptance criterion** | **Key references** |
| --- | --- | --- | --- | --- | --- |
| 1 | bsRNAs in pathogen OMVs carry sequences complementary to the *Smad7* 3'UTR and are compatible with host AGO2 loading | *Smad7* 3′UTR targeting screen plus AGO2-biochemical-compatibility ranking of OMV-sRNA libraries | IntaRNA 2.0, SPOT, small-RNA end analysis, 5′-phosphate-dependent adaptor ligation, RNAfold, OMV/whole-cell RNA comparison | MFE <= -20 kcal/mol, seed complementarity >= 7 nt, and ED < 10 kcal/mol; Class A/B ranking by reproducible 5′/3′ ends and dominant length, 5′ monophosphate, 5′ nucleotide, end structure, and OMV enrichment | [S1]-[S6], [S27], [S29] |
| 2 | Laryngeal opportunistic pathogens release OMVs carrying sRNA cargo under stress | OMV isolation, physical characterization, and small RNA profiling | Density-gradient ultracentrifugation, NTA, TEM, small RNA-seq | MISEV2023-compliant OMVs with detectable 15-50 nt sRNA cargo | [S7]-[S11] |
| 3 | OMV exposure reduces Smad7 expression in VFFs | OMV-VFF co-culture with dose-response and time course | PKH26 uptake tracking, RT-qPCR, Western blot | ≥30% Smad7 protein reduction (Western blot) as the primary criterion; mRNA reduction as a supportive endpoint; RNA-dependent effect (attenuated by heat inactivation) | [S1], [S12]-[S17] |
| 4 | bsRNAs associate with host AGO2/RISC and target the *Smad7* 3′UTR by sequence complementarity | AGO2-RIP, WT/MUT reporter assay, AGO2 knockdown, and AGO2-WT/D669A rescue | AGO2-RIP + RT-qPCR/sRNA-seq; pmirGLO WT/MUT; two AGO2 siRNAs; siRNA-resistant AGO2-WT and AGO2-D669A | >= 5-fold RIP enrichment; >= 50% WT-reporter reduction with no MUT effect; AGO2 knockdown attenuates and AGO2-WT restores the effect; D669A distinguishes slicer dependence | [S1], [S18]-[S24], [S28] |
| 5 | Smad7 silencing drives pro-fibrotic phenotype; rescue reverses it | Fibrotic marker panel; Smad7 overexpression and antagomir rescue | RT-qPCR, Western blot, immunofluorescence, Sirius Red | >2-fold COL1A1/COL3A1; rescued by Smad7 overexpression and antagomir | [S1], [S14], [S25], [S26] |

## References

[S1] Koeppen K, Hampton TH, Jarek M, Scharfe M, Gerber SA, Mielcarz DW, et al. A novel mechanism of host-pathogen interaction through sRNA in bacterial outer membrane vesicles. PLoS Pathog. 2016;12(6):e1005672. doi: 10.1371/journal.ppat.1005672

[S2] Mann M, Wright PR, Backofen R. IntaRNA 2.0: enhanced and customizable prediction of RNA-RNA interactions. Nucleic Acids Res. 2017;45(W1):W435-W439. doi: 10.1093/nar/gkx279

[S3] Raden M, Miladi M. How to do RNA-RNA interaction prediction? A use-case driven handbook using IntaRNA. Methods Mol Biol. 2024;2726:209-234. doi: 10.1007/978-1-0716-3519-3_9

[S4] King AM, Vanderpool CK, Degnan PH. sRNA target prediction organizing tool (SPOT) integrates computational and experimental data to facilitate functional characterization of bacterial small RNAs. mSphere. 2019;4(1):e00561-18. doi: 10.1128/mSphere.00561-18

[S5] Mosca K, Turbant F, Achouak W, Drira M, Debard R, Barakat M, et al. RNAs associated with bacterial outer membrane vesicles: structural insights into surface composition. J Extracell Vesicles. 2026;15(6):e70306.

[S6] Diallo I, Ho J, Lalaouna D, Masse E, Provost P. RNA sequencing unveils very small RNAs with potential regulatory functions in bacteria. Front Mol Biosci. 2022;9:914991. doi: 10.3389/fmolb.2022.914991

[S7] Toyofuku M, Nomura N, Eberl L. Types and origins of bacterial membrane vesicles. Nat Rev Microbiol. 2019;17(1):13-24. doi: 10.1038/s41579-018-0112-2

[S8] Melo J, Pinto V, Fernandes T, Malheiro AR, Osorio H, Figueiredo C, et al. Isolation method and characterization of outer membranes vesicles of Helicobacter pylori grown in a chemically defined medium. Front Microbiol. 2021;12:654193. doi: 10.3389/fmicb.2021.654193

[S9] Welsh JA, Goberdhan DCI, O'Driscoll L, Buzas EI, Blenkiron C, Bussolati B, et al. Minimal information for studies of extracellular vesicles (MISEV2023): from basic to advanced approaches. J Extracell Vesicles. 2024;13(2):e12404. doi: 10.1002/jev2.12404

[S10] Engevik MA, Danhof HA, Ruan W, Engevik AC, Chang-Graham AL, Engevik KA, et al. Fusobacterium nucleatum secretes outer membrane vesicles and promotes intestinal inflammation. mBio. 2021;12(2):e02706-20. doi: 10.1128/mBio.02706-20

[S11] Zhang L, Zhang D, Liu C, Tang B, Cui Y, Guo D, et al. Outer membrane vesicles derived from Fusobacterium nucleatum trigger periodontitis through host overimmunity. Adv Sci (Weinh). 2024;11(47):e2400882. doi: 10.1002/advs.202400882

[S12] Chu Y, Fang Y, Wu H, Cheng L, Chen J. Establishment and characterization of immortalized human vocal fold fibroblast cell lines. Biotechnol Lett. 2023;45(3):347-355. doi: 10.1007/s10529-023-03350-6

[S13] Gong T, Zhang C, Kang J, Lamb JJ, Jiang JJ. Cryotherapy has antifibrotic and regenerative effects on human vocal fold fibroblasts. Laryngoscope. 2019;129(4):E143-E150. doi: 10.1002/lary.27499

[S14] Chen X, Thibeault SL. Response of fibroblasts to transforming growth factor-beta1 on two-dimensional and in three-dimensional hyaluronan hydrogels. Tissue Eng Part A. 2012;18(23-24):2528-2538. doi: 10.1089/ten.TEA.2012.0094

[S15] Mehanny M, Koch M, Lehr CM, Fuhrmann G. Streptococcal extracellular membrane vesicles are rapidly internalized by immune cells and alter their cytokine release. Front Immunol. 2020;11:80. doi: 10.3389/fimmu.2020.00080

[S16] Rubio APD, Martinez J, Palavecino M, Fuentes F, Lopez CMS, Marcilla A, et al. Transcytosis of Bacillus subtilis extracellular vesicles through an in vitro intestinal epithelial cell model. Sci Rep. 2020;10(1):3120. doi: 10.1038/s41598-020-60077-4

[S17] Han EC, Choi SY, Lee Y, Park JW, Hong SH, Lee HJ. Extracellular RNAs in periodontopathogenic outer membrane vesicles promote TNF-alpha production in human macrophages and cross the blood-brain barrier in mice. FASEB J. 2019;33(12):13412-13422. doi: 10.1096/fj.201901575R

[S18] Bellissimo T, Masciarelli S, Poser E, Genovese I, Del Rio A, Colotti G, et al. Small molecules targeting the miRNA-binding domain of Argonaute 2: from computer-aided molecular design to RNA immunoprecipitation. Methods Mol Biol. 2017;1517:211-221. doi: 10.1007/978-1-4939-6563-2_15

[S19] Angart PA, Adu-Berchie K, Carlson RJ, Vocelle DB, Chan C, Walton SP. Relative quantification of siRNA strand loading into Ago2 for design of highly active siRNAs. Methods Mol Biol. 2019;1974:41-56. doi: 10.1007/978-1-4939-9220-1_4

[S20] Li WX, Ding SW. Mammalian viral suppressors of RNA interference. Trends Biochem Sci. 2022;47(11):978-988.

[S21] Wang C, Sheng W, Zhou Y, Gao Y, Wang K, Liu R, et al. siRNA-AGO2 complex inhibits bacterial gene translation: a promising therapeutic strategy for superbug infection. Cell Rep Med. 2025;6(3):101997.

[S22] Panwalkar P, Khire A, Shirsat N. Validation of microRNA target genes using luciferase reporter assay and Western blot analysis. Methods Mol Biol. 2022;2423:27-38. doi: 10.1007/978-1-0716-1952-0_3

[S23] Abel Y, Rederstorff M. Gene reporter assays to study miRNA function. Methods Mol Biol. 2021;2300:119-131. doi: 10.1007/978-1-0716-1386-3_12

[S24] Choi C, Han J, Thao Tran NT, Yoon S, Kim G, Song S, et al. Effective experimental validation of miRNA targets using an improved linker reporter assay. Genet Res (Camb). 2017;99:e2. doi: 10.1017/S001667231600015X

[S25] Branco A, Bartley SM, King SN, Jette ME, Thibeault SL. Vocal fold myofibroblast profile of scarring. Laryngoscope. 2016;126(3):E110-E117. doi: 10.1002/lary.25581

[S26] Liu D, Qian T, Li P, Zheng Z, Yang Y, Liu X, et al. Asiatic acid improves extracellular matrix remodeling in vocal fold scarring via SMAD7 activation. Laryngoscope. 2022;132(6):1237-1244.

[S27] Frank F, Sonenberg N, Nagar B. Structural basis for 5′-nucleotide base-specific recognition of guide RNA by human AGO2. Nature. 2010;465(7299):818-822. doi: 10.1038/nature09039

[S28] Broderick JA, Salomon WE, Ryder SP, Aronin N, Zamore PD. Argonaute protein identity and pairing geometry determine cooperativity in mammalian RNA silencing. RNA. 2011;17(10):1858-1869. doi: 10.1261/rna.2778911

[S29] Nakanishi K. Anatomy of four human Argonaute proteins. Nucleic Acids Res. 2022;50(12):6618-6638. doi: 10.1093/nar/gkac519
